# MACE: model based analysis of ChIP-exo

**DOI:** 10.1093/nar/gku846

**Published:** 2014-09-23

**Authors:** Liguo Wang, Junsheng Chen, Chen Wang, Liis Uusküla-Reimand, Kaifu Chen, Alejandra Medina-Rivera, Edwin J. Young, Michael T. Zimmermann, Huihuang Yan, Zhifu Sun, Yuji Zhang, Stephen T. Wu, Haojie Huang, Michael D. Wilson, Jean-Pierre A. Kocher, Wei Li

**Affiliations:** 1 Division of Biomedical Statistics and Informatics, Mayo Clinic, Rochester, MN 55905, USA; 2 Division of Biostatistics, Dan L. Duncan Cancer Center and Department of Molecular and Cellular Biology, Baylor College of Medicine, Houston, TX 77030, USA; 3 School of Life Science and Technology, Tongji University, Shanghai 200092, China; 4 Genetics & Genome Biology Program, SickKids Research Institute, 686 Bay St. Toronto, ON, M5G 0A4, Canada; 5 Department of Biochemistry and Molecular Biology, Mayo Clinic, MN 55905, USA; 6 Department of Molecular Genetics, University of Toronto, Toronto, Ontario M5S1A8, Canada

## Abstract

Understanding the role of a given transcription factor (TF) in regulating gene expression requires precise mapping of its binding sites in the genome. Chromatin immunoprecipitation-exo, an emerging technique using λ exonuclease to digest TF unbound DNA after ChIP, is designed to reveal transcription factor binding site (TFBS) boundaries with near-single nucleotide resolution. Although ChIP-exo promises deeper insights into transcription regulation, no dedicated bioinformatics tool exists to leverage its advantages. Most ChIP-seq and ChIP-chip analytic methods are not tailored for ChIP-exo, and thus cannot take full advantage of high-resolution ChIP-exo data. Here we describe a novel analysis framework, termed MACE (model-based analysis of ChIP-exo) dedicated to ChIP-exo data analysis. The MACE workflow consists of four steps: (i) sequencing data normalization and bias correction; (ii) signal consolidation and noise reduction; (iii) single-nucleotide resolution border peak detection using the Chebyshev Inequality and (iv) border matching using the Gale-Shapley stable matching algorithm. When applied to published human CTCF, yeast Reb1 and our own mouse ONECUT1/HNF6 ChIP-exo data, MACE is able to define TFBSs with high sensitivity, specificity and spatial resolution, as evidenced by multiple criteria including motif enrichment, sequence conservation, direct sequence pileup, nucleosome positioning and open chromatin states. In addition, we show that the fundamental advance of MACE is the identification of two boundaries of a TFBS with high resolution, whereas other methods only report a single location of the same event. The two boundaries help elucidate the *in vivo* binding structure of a given TF, e.g. whether the TF may bind as dimers or in a complex with other co-factors.

## INTRODUCTION

Precise and comprehensive mapping of transcription factor (TF) binding sites (TFBSs) is important for understanding the mechanisms that regulate gene regulation. Chromatin immunoprecipitation (ChIP) is the most widely used approach to study *in vivo* protein–DNA interactions (1). In a cross-linked ChIP assay, proteins are cross-linked to their target DNA and then immunopurified from sheared chromatin. After reversing the cross-links, protein bound DNA fragments are subjected to microarray hybridization (ChIP-chip) or deep sequencing (ChIP-seq). Owing to the rapid technological developments in high-throughput sequencing, ChIP-chip is less commonly used because of its lower resolution, smaller dynamic range, limited sensitivity and increased cost when applied to the entire mammalian genome (2–4).

ChIP-seq is the most popular technique for TFBS mapping and provides higher resolution than ChIP-chip. However, its resolution is still highly limited by chromatin shearing via sonication that breaks ChIPed DNA into a fixed fragment size (e.g. 200 bases), which is much bigger than the actual TFBS (e.g. 6–20 bases). To overcome this limit, Rhee *et al.* developed ChIP-exo, a technique that uses λ phage exonuclease to digest the 5′ end of TF-unbound DNA after ChIP (4). In ChIP-exo, λ exonuclease digestion leaves homogenous 5′ ends of DNA fragments at the actual two boundaries of TFBS, and after sequencing and mapping reads to the reference genome, two borders of TFBS could be defined. The λ exonuclease treatment also helps eliminate contaminating DNA and increases the signal-to-noise ratio (SNR), which enables the identification of true albeit weak bindings. Overall, ChIP-exo provides a direct, unbiased and near–single base pair (bp) resolution mapping of protein–DNA interactions *in vivo*, and promises new insights into TFBS mapping and gene regulation.

Numerous tools have been developed to analyze ChIP-chip and ChIP-seq data (5–14). However, most of them cannot fully leverage the advantages of ChIP-exo. First, ChIP-exo data is different from that of ChIP-seq because of the λ exonuclease treatment, which trims unbound DNA to almost the same positions (i.e. the borders of TFBSs). As a result, multiple reads aligning to the identical genomic position (a.k.a. clonal reads) should be handled differently. In ChIP-seq, clonal reads are likely to be originated from polymerase chain reaction (PCR) over amplification and should be properly depleted, whereas in ChIP-exo, clonal reads are expected because of the selection of λ exonuclease digestion. Second, TFBS detection is conceptually different in the two methods. In ChIP-seq, a region is reported as a candidate TFBS if its coverage signal is significantly higher than background, whereas ChIP-exo can define two boundaries of the same TFBS, by virtue of the directional digestion of λ exonuclease. While several computational tools have been successfully applied to predict TFBSs using ChIP-exo data, these methods were originally designed for ChIP-seq, and thus did not exploit the unique advantages of ChIP-exo (15,16).

Here we introduce model-based analysis of ChIP-exo (MACE), a novel computational tool taking advantage of the unique characteristics of ChIP-exo data. MACE includes the following four major steps: (i) sequencing data normalization and bias correction; (ii) signal consolidation and noise reduction; (iii) single-nucleotide resolution border peak detection using the Chebyshev Inequality and (iv) border matching using the Gale–Shapley stable matching algorithm (see ‘Materials and Methods’). We applied MACE to both published and our own ChIP-exo datasets and evaluated its performance using the cognate DNA motif, sequence conservation, nucleosome position (MNase-seq), DNA accessibility (FAIRE-seq and DNase-seq) and ENCODE ChIP-seq data. Our results indicate that MACE can identify real TFBSs with high sensitivity, specificity and spatial resolution.

## MATERIALS AND METHODS

### Public data resources

We downloaded human CCCTC-binding factor (CTCF) and yeast Reb1 ChIP-exo data from the NCBI Short Read Archive (http://www.ncbi.nlm.nih.gov/sra; accession number SRA044886) (4). We downloaded human nucleosome data from the NCBI Gene Expression Omnibus (http://www.ncbi.nlm.nih.gov/geo; accession number GSE26501) (17). Yeast nucleosome data were generated by Xi *et al.* and downloaded from Gene Expression Omnibus (accession number GSE26412) (18). Human DNase-seq, FAIRE-seq and CTCF ChIP-seq data were generated by the ENCODE consortium and downloaded from the UCSC genome browser (http://genome.ucsc.edu/) (19). We aligned human CTCF ChIP-exo raw reads to GRCh37/hg19 and yeast Reb1 ChIP-exo raw reads to SacCer3 using Bowtie (20). Mouse HNF6 ChIP-exo raw reads were aligned to mouse reference genome (mm10/GRCm38). Only the unique alignments were used for downstream analysis. PhastCon conservation scores between 46 vertebrate genomes and between seven yeast species were downloaded from the UCSC table browser. *In silico* predictions of CTCF and Reb1 binding sites were performed by Find Individual Motif Occurrences (FIMO), using a *P*-value cutoff of 1E-6 (21). Position-weighted matrices of CTCF and Reb1 motifs were retrieved from the Jaspar database (http://jaspar.genereg.net/) (22).

### Mouse ONECUT1 ChIP-exo data

For each of three biological ChIP-exo replicates, post mortem livers from three male C57BL/6J mice, ∼4 months old, were used. These mice were maintained in specific pathogen-free conditions at the Hospital for Sick Children Laboratory Animal Services according to an approved animal use protocol and were kindly provided by Dr Jayne Danska. Approximately one-third of each liver was utilized per ChIP-exo experiment. Seven microgram of an antibody against the liver-enriched TF ONECUT1 (HNF6; Santa Cruz Biotechnology antibody sc13050) was used for each biological replicate. Livers were cross-linked in 1% formaldehyde and ChIP was performed as previously described (up until and including the RIPA buffer washes; step 38 from Schmidt *et* *al*. 2009) (23). The ChIP-exo portion of the protocol was performed as previously described (Rhee and Pugh 2011) with modifications making the assay compatible with the Illumina sequencing platform. Briefly, while still on the magnetic beads (Invitrogen, Dynabeads), the immunoprecipitated DNA was end-repaired (NebNext^®^ End Repair Module, New England Biolabs (NEB)). The P7 adapter (150 pmol) was designed based on the adapter sequence provided by NEB: 5′-Phos-TGACTGGAGTTCAGACGTGTGCTCTTCCGATCT-OH-3′ and 5′-OH-AGATCGGAAGAGCACACGTCTGAACTCC-OH-3′. After ligation, the DNA was nick-repaired with phi29 polymerase, and digested by lambda (λ) and RecJf exonucleases (NEB). DNA samples were eluted from the beads by performing reverse cross-linking overnight at 65°C, followed by RNaseA (Ambion) and ProteinaseK (Invitrogen) treatments. DNA was extracted using a phenol-chloroform-isoamyl protocol and ethanol precipitation. DNA was denatured at 95°C, and 3′ ends were primer-extended with a P7 fill-in primer (5 pmol; 5′-OH-TGACTGGAGTTCAGACGTGTGCTCTTCCGATCT-OH-3′) and phi29 polymerase. A second adaptor ligation to only λ exonuclease-digested ends of double stranded DNA was performed using 15 pmol of P5 adapter (sequence provided by NEB: 5′OH-AGATCGGAAGAGCGTCGTGTAGGGAAAGAGTG-OH-3′ and 5′-OH-TCTACACTCTTTCCCTACACGACGCTCTTCCGATCT-OH-3′). The resulting DNA samples were PCR-amplified using multiplexing index primers (NEBNext^®^ DNA Library Prep Master Mix Set for Illumina^®^). Libraries of 180–300 bp were electrophoretically size selected with a 2% Pippin Prep gel (Sage Science), quantified with 2100 Bioanalyzer (Agilent) and 50 bp reads were sequenced with the HiSeq2500 (Illumina) by the Donnelly Sequencing Centre, Toronto. All raw data is publicly available in ArrayExpress under E-MTAB-2060.

### Sequencing data normalization and bias correction

To define two borders of a TFBS with single-nucleotide resolution, we processed reads mapped to forward and reverse DNA strands separately, and only the coverage signals contributed by the 5′ end of reads were used for downstream analysis. Because sequencing depth may vary significantly between samples, we used a size factor (*F_j_*) to normalize sequencing depth to a common scale (e.g. 10 million total reads):

\begin{equation*} \hat F_{j} = \frac{{1.0 \times 10^7 }}{{T_{j} }} \end{equation*}

where *T_j_* is the total number of reads mapped to unique locations to the reference genome in sample *j*.

Bias in nucleotide composition (i.e. dependence of nucleotide frequency on position of the read) has been reported in both RNA-seq and DNA-seq (24). We observed similar bias from multiple independent ChIP-exo datasets from different groups using different sequencing platforms (Illumina^®^ and SOLiD^®^), suggesting that the prevalence of this bias in all ChIP-exo sequencing (Supplementary Figure S1A–C). Such bias not only impacts the coverage uniformity, but more importantly, it affects TFBS border peak detection. We first estimated the background nucleotide composition bias from ‘singleton reads’ (i.e. reads that can be uniquely mapped to the genome but have no overlap with any other reads). The purpose of using singleton reads is to preclude confounding factors such as PCR, IP or exonuclease selection. Assuming the frequency of the first k-mer of reads was independent and identically distributed, bias occurred if a k-mer frequency was much larger or smaller than 1/(4^k^). Hansen *et* *al*. proposed a weighting function to correct such bias by assigning each read a weight based on its first heptamer (24). Although first proposed in RNA-seq data, the weighting scheme can be adapted to correct any sequencing bias occurring at the beginning of reads. The weight for a particular oligomer (*h*) is calculated as:

\begin{equation*} W(h) = \frac{{\frac{1}{{L - h}}\sum\limits_{n = 1}^{L - h} {\hat P_n (h)} }}{{\hat P_0 (h)}} \end{equation*}

where *h* represents oligomer (default hexamer or 6-mer), *L* is length of read; $\hat P_0 (h)$ is the proportion of reads with *h*-mer at the beginning (5′ end); and $\hat P_n (h)$ is the proportion of reads with *h*-mer starting at *n*th position (*n* = 1, 2,…, *L − h*). For reads without positional bias $\hat P_0 (h)$ is close to the mean of $\hat P_n (h)$, and therefore *W*(*h*) ≈ 1.

### Signal consolidation and noise reduction

Real ChIP-exo signals are usually confounded by factors such as inexact cross-linking, exonuclease digestion, dynamic conformations of protein and PCR amplification. Signals generated from such undesirable factors are nonspecific and can be greatly reduced if we consolidate multiple replicates. We used Shannon's (relative) entropy (*H*) to consolidate replicates signal because: (i) it automatically considers biological variance between replicates; large variance indicates poor reproducibility and therefore has lower entropy score; (ii) perfect reproducibility will maximize Shannon's entropy; (iii) its logarithmic scale will help reduce hyper dispersion incurred in ChIP-exo data; (iv) a larger number of replicates will have higher entropy. This is useful when a particular replicate has no coverage (i.e. zero read count) at a particular position in the genome. For instance, Shannon's entropy is 1.386 in the case of perfect reproducibility among four replicates; however, if one replicate has no coverage at this position, entropy decreases to 1.099. This decrease is appropriate because confidence decreases when no signal was detected at certain sequencing depth. Finally, (v) it is straightforward to scale entropy (from 0 to 1) because the maximum entropy is fixed for a given number of replicates. The consolidated signal *S_i_* of all replicates (indexed by *j*) at each nucleotide position (indexed by *i*) was calculated as follows:

\begin{equation*} S_{{i}({\rm Entropy})} = \frac{1}{n}\sum\limits_{j = 1}^n {C_{{ij}} F_{\rm j} W_{i} \times \left( {\frac{{\sum\limits_{j = 1}^n {p_{j} \times \log \,p_{j} } }}{{\sum\limits_1^n {\frac{1}{n} \times \log \,\frac{1}{n}} }}} \right)} \end{equation*}

where *C_ij_* is the raw coverage derived from the 5′ end of reads at a position *i* in replicate *j*; *n* is the number of replicates (*j* = 1,…,*n*); *F_j_* is the size factor used to normalize sequencing depth defined in previous section; *W_i_* is the weight to correct nucleotide composition bias defined in previous section; and *p_j_* is the proportion of reads belonging to replicate *j* at a particular position. *P_j_* is calculated independent of *i* (a particular genome position). In other words, we will calculate relative entropy for each nucleotide position of a particular locus. Basically, *S_i_* is the raw coverage (*C_ij_*), normalized by library size (*F_j_*) to correct sequencing depth, then weighted by starting *k*-mer frequency (*Wi*) to correct nucleotide composition bias, and then weighted by *relative entropy* to consolidate and measure reproducibility between replicates.

We compared this entropy-based noise reduction scheme to other methods including arithmetic mean (AM), geometric mean (GM) and SNR. These methods are defined as follows:

\begin{equation*} \begin{array}{*{20}l} {S_{{i}({\rm AM})} = \frac{1}{n}\sum\limits_{j = 1}^n {C_{{ij}} F_{j} W_{i} ;\,S_{{i}({\rm GM})} = \left( {\prod\limits_{j = 1}^n {C_{{ij}} F_{j} W_{i} } } \right)^{{{\scriptstyle 1} \kern-0.1em/\kern-0.15em {\scriptstyle n}}} ;} } \\ {S_{{i}({\rm SNR})} = \frac{\mu }{\sigma } = \frac{{{\rm Mean}(C_{{ij}} F_{j} W_{i} )}}{{{\rm Stdev}(C_{{ij}} F_{j} W_{i} )}}} \\ \end{array} \end{equation*}

### Border peak detection

It is expected that coverage signals at TFBS boundaries are significantly higher than flanking regions. Therefore, border peak detection is essentially to identify ‘outlier’ sites with unusually high coverage. Numerous sophisticated approaches for outlier detection are available, but many are limited in that they assume a distribution or require predefined upper and lower boundaries. We chose a nonparametric method based on the Chebyshev's inequality because (i) it makes no assumptions about the distribution of the coverage signals; (ii) it assumes that a relatively small percentage of outliers are included in the data (in ChIP-exo experiments, we also expect that only a few positions are real borders in a TFBS); and (iii) this method is computationally efficient and statistically robust. The Chebyshev Inequality states that for a random variable *X* with finite mean *μ* and nonzero variance *σ^2^* and for any real number *k* > 1 (the inequality becomes vacuous when *k* ≤ 1):

\begin{equation*} \Pr (|X - \mu | \ge k\sigma ) \le \frac{1}{{k^2 }} \end{equation*}

Applying this into border peak detection and using *m* and *s* as estimators of *μ* and *σ*. *m* and *s* are the average and standard deviation of ChIP-exo signal within a user specified, local genomic interval (or background region), we have:

\begin{equation*} \Pr (X - m \ge ks) \le \frac{1}{{k^2 }} \end{equation*}

For example, if the coverage at a particular position (*i*) in a particular genome interval was 4.5 SDs (standard deviations) larger than the mean (*m*), then the associated pseudo *P*-value for this candidate border is 1/(4.5**2) ≈ 0.05. In other words, within this genomic interval, the chance to observe a value that is the same or larger than that of position *i* is 0.05. Similar to the ‘local lambda’ in MACS that captured the influence of local biases, *m* and *s* reflected the local average signal and variability (7). As a non-parametric method, Chebyshev Inequality is robust but very conservative. However, in most cases, we expect only two border peaks out of a protein-binding site, and therefore we need the border peak detection method as conservative as possible. Too many border peaks will make the downstream ‘border matching’ step very difficult, and produce a large number of false positive border pairs. Although this theorem provides upper bounds rather than real *P*-values, the Chebyshev Inequality remains one of the optimal solutions for outlier detection (25).

### Border matching

After border peak detection, the next step is to identify border pairs that can demarcate TFBSs. According to the ChIP-exo protocol, one forward border need to pair with one downstream reverse border and such pairings should be done in an exclusive manner (i.e. one border can only be included in one pair). Because a given TFBS may have multiple forward and reverse candidate borders derived either from multiple cross-linking positions or spurious noise, exhaustive matching (trying all possible pairings between forward and reverse borders) could produce excessive false-positives. Approaches such as matching a forward border to its nearest reverse border or matching the two borders with the highest signal may work for TFBSs with higher sequencing depth and fewer or no spurious borders. We rendered border matching as a stable matching problem that could be solved by using classic algorithms such as the Gale-Shapley algorithm (26). The stability of matching is always guaranteed with this algorithm.

By assuming that the sizes of TFBS is relatively stable throughout the genome, we can first estimate the optimal size and then use the optimal size as the standard to weight all candidate border pairs within a location. The quality of the border pair is determined primarily by two factors­: coverage score *S* (i.e. coverage intensity) and border-pair size *d* ($d\sim N(\mu _{\rm T} ,\sigma _{\rm T} )$); an optimal border pair would maximize *S*, and would be close to the biological expectation. Therefore, before applying the Gale–Shapley algorithm to perform border matching, we need to weight coverage score (*S*) with distance (*d*) and penalize the border-pairs with distances that are unusually larger or smaller than expectation. We used the following weighting function:

\begin{equation*} S_{{\rm weighted}} = S_{{\rm obs}} \times w = S_{{\rm obs}} \times \exp \left( { - \frac{{(d_{{\rm obs}} - \mu _{\rm T} )^2 }}{{K^2 }}} \right) \end{equation*}

where *S*_obs_ is the observed coverage score and *S*_weighted_ is the coverage score weighted by *d*; *μ*_T_ is the mathematic expectation of *d* and can be empirically estimated from a small subset of high-confidence border pairs (i.e. borders that can be unambiguously matched to each other). We used Gaussian mixture models to estimate *μ*_T_, because the distribution of border pair size usually exhibits more than one mode (see Figure 4 in Results). We assigned arbitrary initial values to *μ*_T_ and then used an expectation maximization algorithm to iteratively refine this value until it converged. *K* is kernel width indicating weighting magnitude; a larger *K* value indicates a smaller impact of *d* on *S*_weighted_.

We incorporated the Gale–Shapley algorithm into the border-pairing optimization procedure. Briefly, in a particular binding site, each border on the forward strand (*f_i_*, *i* = 1, 2, 3…) will search for its candidate partners from the reverse stand (*r_j_*, *j* = 1, 2, 3…) based on its own preference order, and vice versa. The Gale–Shapley algorithm generates optimized, stable border pair(s). All matches are stable when there does not exist any alternative pairings in which both *f_i_* and *r_j_* are better off than they would be with the element they are currently matched. Because λ exonuclease digestions on two borders are independent events, we then determined the significance level of border pairs using the Fisher method to combine *P*-values (*P*_border-1_ and *P*_border-2_) of two borders into a single border-pair *P*-value (*P*_borderpair_):

\begin{eqnarray*} &&P_{{\rm borderpair}}\nonumber\\ &&= P_{{\rm border} - 1} \times P_{{\rm border} - 2} \times (1 - \log (P_{{\rm border} - 1} \times P_{{\rm border} - 2} )) \end{eqnarray*}

## RESULTS

### Flowchart of the MACE algorithm

**Figure 1.**
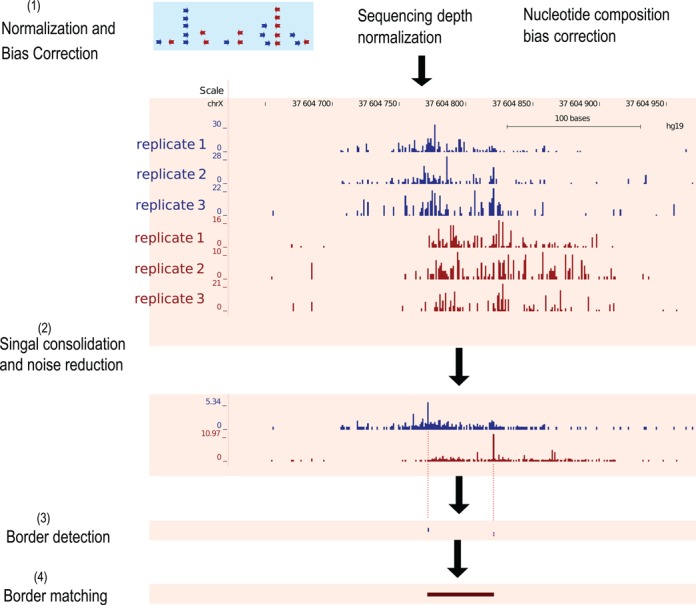
MACE's working procedure. (1) Sequencing depth normalization and nucleotide composition bias correction. (2) Replicate consolidation and noise reduction. Coverage signals were represented in BigWig format to facilitate visualization and downstream processing. Reads mapped to forward and reverse strands were processed separately, and only the 5′ ends of reads were used. Shown here were forward (blue) and reverse (red) coverage signals calculated from three biologic replicates. (3) Border peak detection. Significance of border peaks was determined using the Chebyshev inequality. (4) Border matching using the Gale–Shapley stable matching algorithm.

MACE processes ChIP-exo data in four steps: (i) sequencing data normalization and bias correction; (ii) signal consolidation and noise reduction; (iii) single-nucleotide resolution border peak detection using the Chebyshev Inequality; and (iv) border matching using the Gale–Shapley stable matching algorithm (Figure 1). We evaluated the computation performance of MACE using a single Intel Xeon CPU (2.67 GHz). It consumed <1 Gb RAM and required about 10 h to process human CTCF ChIP-exo data that had three replicates with a total of 82 million aligned reads.

### Nucleotide composition bias correction

We first estimated the distribution of nucleotide composition bias from singleton reads, then corrected the bias by reweighting each read according to its first K-mer. Using CTCF ChIP-exo data as an example, we demonstrated that this sequencing bias was effectively eliminated at the sequence level (Supplementary Figure S1C and D). To evaluate the impact of bias correction on border definition, we selected 5000 *in silico* predicted, high-confidence CTCF motifs and calculated the coverage profile around them. Although there was no obvious improvement on the definition of four major borders (F1, F2, R1, R2, see below), spurious signals within motif regions (between two vertical dashed lines) were indeed reduced after correction (Supplementary Figure S2).

We next investigated whether this nucleotide composition bias correction improved the overall performance of MACE. We ran MACE without the bias correction step and compared results to those obtained from a complete MACE run. We found that nucleotide composition bias correction seemed to have little effect on the final number of border pairs detected; we identified 41 407 unique border pairs (52 616 with redundancy) with 90.12% (compared to 90.09% obtained from complete MACE run) of them could be verified by ChIP-seq results from ENCODE (Supplementary Figure S3A), and 42.78% (compared to 43.72% obtained from complete MACE run) of them contain canonical CTCF motif (Supplementary Figure S3B). However, the distance between border pair and motif is indeed increased (i.e. the spatial resolution becomes worse) without bias correction (Supplementary Figure S3C). Overall there is limited improvement by performing nucleotide composition bias correction, therefore, this step (nucleotide bias correction) is optional and can be turned off in MACE analysis procedure.

### Noise reduction

**Figure 2.**
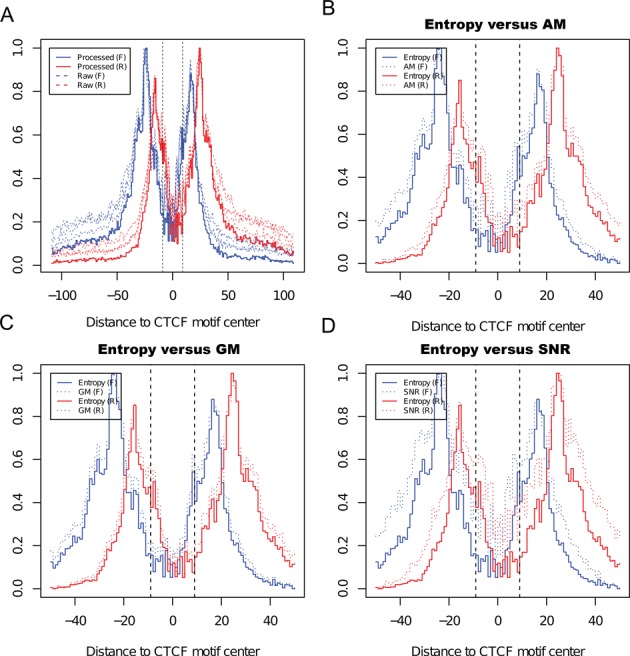
Compare noise reduction effect using different methods. A, Shannon entropy based noise reduction effect illustrated by coverage profile around predicted CTCF motif. Forward and reverse coverage signals (y-axis) were normalized and displayed using blue and red curves, respectively. Dashed curves indicated raw signals before noise reduction (each curve represented 1 replicate), and solid curves indicated consolidated signals after noise reduction. B, C and D, Comparing entropy based noise reduction scheme to AM, GM and SNR. In panels B, C and D, signals processed with entropy were indicated using solid curve, and signals processed with AM, GM, SNR were indicated using dashed curves.

To assess the effects of noise reduction, we selected 5000 *in silico* predicted CTCF motifs as we did previously and calculated the coverage profile around them. Raw signals (dashed curves) far from the CTCF motif were dramatically reduced to a lower level after entropy-based noise reduction (solid curves) (Figure 2A). As an example, we chose a binding site located in the promoter region of *Myc*, a well-known target gene of CTCF. As expected, spurious signals were significantly reduced and two boundaries of the binding site could be clearly detected. The binding site delimited by the identified border pair was much smaller than the region covered by the raw signal. More importantly, the binding site was highly conserved and centered on the predicted CTCF motif, suggesting the authenticity of this border pair (Supplementary Figure S4A).

Despite the advantages of Shannon's entropy in combining biological replicates and reducing spurious signals (see ‘Materials and Methods’), we have not shown whether it is better than other commonly used simple methods. To answer this question, we compared entropy-weighted average with AM, GM and SNR using the same 5000 motifs as above. Because scores (*S*) calculated from these methods were in different scale, we normalized all scores between 0 and 1 using *S′* = (*S* − *S*_min_)/(*S*_max_ − *S*_min_). Our results indicated that the entropy-weighted average outperformed all other methods in measuring reproducibility and reducing noisy signals (Figure 2B–D).

We further investigated the performance of the entropy-based noise reduction method on binding sites with relatively weak signals. We ranked predicted CTCF motifs in descending order according to the ChIP-exo tag intensity and then divided them equally into four groups: the first quantile (0–25%, strongest binding), the second quantile (25–50%, modestly strong binding), the third quantile (50–75%, modestly weak binding) and the fourth quantile (75–100%, weakest binding). By comparing the signal profile before and after noise reduction, we found that the entropy-based noise reduction strategy was most useful when the ChIP-exo signal became weaker (Supplementary Figure S5). This is because weaker bindings have relatively higher level of noise (i.e. lower SNR), and therefore the room for improvement is larger. This further highlighted the effectiveness and robustness of entropy-based noise reduction method.

To explore how entropy-based noise reduction scheme impacts the final border pair detection, we ran MACE without the noise reduction step. Compared to the complete MACE run, we detected 12% fewer border pairs (36 355 versus 41 222). More importantly only 82.42% of them could be verified by ENCODE ChIP-seq results, compared to 90.08% obtained from the complete MACE run (Supplementary Figure S3A). This underlined the indispensability of noise reduction during ChIP-exo analysis.

### Identification of Reb1 binding sites in yeast genome

**Figure 3.**
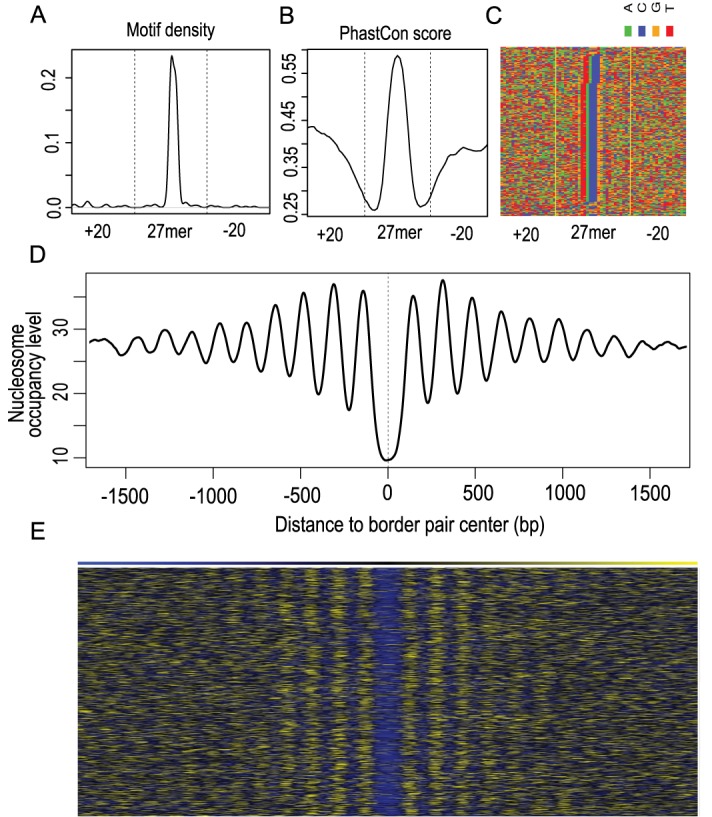
Evaluation of identified Reb1 border pairs using DNA motif, conservation and nucleosome position. (**A**) Reb1 motif (TTACCC[G/T]) density profile (y-axis) over all 27mer border pairs. (**B**) Conservation profile (y-axis) over all 27mer border pairs. Sequence conservation was measured by phastCon score calculated by UCSC from multiple alignments of seven yeast genomes. (**C**) Direct pileup of DNA sequences of all 27mer, and nucleotides were illustrated with different colors: A (green), C (blue), G (orange) and T (red). Sequences were sorted by Reb1 motif position to facilitate visualization. (**D**) Nucleosome occupancy profile (y-axis) showing oscillatory pattern around the midpoint of border pairs. All detected border pairs first were aligned by midpoints (x-axis) and extended 1.5 kb upstream and downstream; MNase-seq tag density then was calculated for the 3-kb windows. (**E**) Heatmap showing nucleosome positions around border pair midpoints. Yellow and blue indicate higher and lower nucleosome occupancy levels, respectively.

To demonstrate the performance of MACE, we applied MACE to yeast Reb1 ChIP-exo data published by Rhee *et al.*, we identified 1192 bp; the most frequent border-pair lengths are 26 (21.8%) and 27 bp (22.3%) (Supplementary Figure S6A, Supplementary Table S1) (4). When searching for the canonical Reb1 motif (TTACCCK) in a 61-bp window (e.g. extending 30 bp upstream and downstream to the middle point of the border pair), we found 1118 border pairs (94%) encompassing the Reb1 motif, and the majority of border pairs were localized in promoter regions that were 158 bp upstream of the transcription start site (Supplementary Figure S7). To evaluate the accuracy of detected binding sites, we picked 26mer and 27mer border pairs (because they were most frequent) and performed motif searching. As demonstrated by the motif density profiles, Reb1 motifs were highly concentrated in the center of border pairs (Figure 3A, C and Supplementary Figure S6B and D).

We questioned whether cis-regulatory elements such as Reb1 binding sites were highly conserved across species. To test this, we calculated the average phastCon conservation score derived from seven yeast species for the 26mer and 27-mer border pairs mentioned above. In concordance with the motif density profile, the border pair center has the highest phastCon score (Figure 3B and Supplementary Figure S6C). Probably the most apparent evidence is to overlay DNA sequences of border pairs directly, as shown by multiple studies (4,27,28). Note that border-pair pileup is different from motif-centered alignment performed by Rhee *et al.*, in that motifs are guaranteed to align together using the latter approach. As shown in Figure 3C and Supplementary Figure S6D, Reb1 motifs were still visually recognized and located exactly in the middle when piling up MACE-determined border pairs.

Because Reb1 has an essential role in organizing chromatin and phasing flanking nucleosomes, we explored the relationship between the identified Reb1 border pairs and nucleosome occupancy (29,30). We lined up all Reb1 border pairs by their midpoints and extended by 1.5 kb upstream and downstream, and calculated nucleosome occupancy measured by tag density from MNase-seq (18). We found that Reb1 border pairs were located exactly in nucleosome-free regions (NFRs) and the surrounding ∼20 nucleosomes were well positioned (Figure 3D and E). This further demonstrated the authenticity and accuracy of Reb1 border pairs as detected by MACE. Altogether, we exemplified the accuracy of MACE in identification of TFBSs from ChIP-exo data in the yeast genome.

### Identification of CTCF binding sites in the human genome

We next applied our method to human CTCF ChIP-exo data published by Rhee *et al.*, (Supplementary Table S2) (4). The distribution of raw tags surrounding CTCF motifs showed four peaks (denoted as F1, F2, R1 and R2), which marked the four exonuclease-derived borders. Rhee *et al.* proposed that the outer borders (F1, R1) and inner borders (F2, R2) reflected two different ‘stops’ of exonuclease. It may also have resulted from different zinc finger usage by CTCF (31). We observed a similar pattern in yeast Reb1 ChIP-exo data, suggesting that this may be a common pattern for ChIP-exo data (Supplementary Figure S8). These four peaks could define four possible borders pairs (F1–R1, F1–R2, F2–R1 and F2-R2). Considering the possibility that spurious signals and protein cofactors would generate more additional ‘confounding borders’, the real situation would be more complicated.

**Figure 4.**
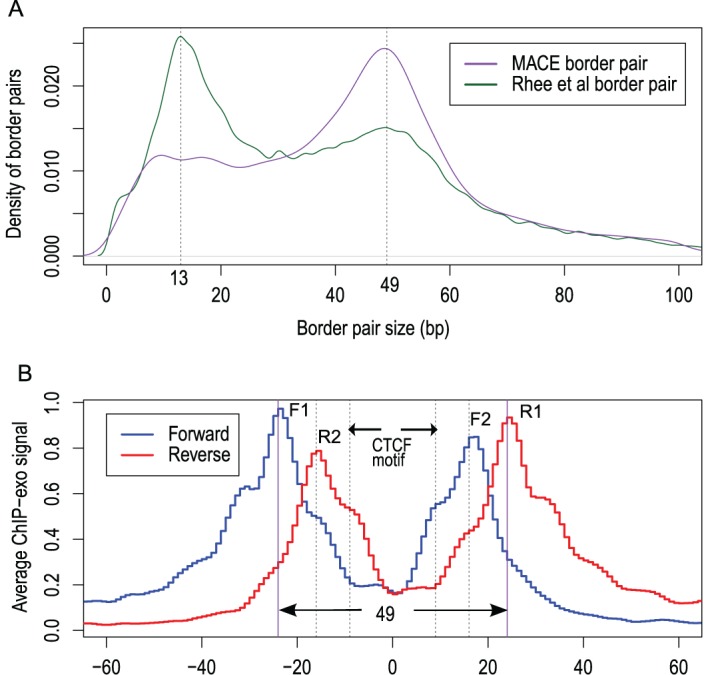
(**A**) Size distribution of border pairs called by MACE (purple) and by Rhee *etal*. (2011) (green). MACE border pair had a single mode (centered at 49), whereas border pairs identified by Rhee *etal*. had bimodal distribution (centered at 13 and 49). (**B**) CTCF ChIP-exo raw sequencing tags density profile around predicted CTCF motifs. The blue and red curve indicates the tag density profile calculated from reads mapped to the forward and reverse strand, respectively. Bimodal patterns were observed for forward and reverse signals.

We applied the Gale–Shapley stable matching algorithm to find the optimal matches between forward and reverse borders (see ‘Materials and Methods’). Strikingly, most border pairs identified by MACE were 49 bp—exactly the same size of F1-R1 as estimated from the unbiased, raw sequencing tag profile (Figure 4A and B), demonstrating the power of our border-matching algorithm. In contrast, binding sites identified by Rhee *et al.* (Supplementary Figure S6 in Rhee *et al.*) exhibited a bimodal distribution with a major mode centered on 13 bp and a minor mode on 49 bp, the 49-bp mode corresponded to F1-R1 matches mentioned above, and the 13-bp mode probably represented F1-R2 or F2-R1 matches that were paired using ‘closest principle’ (Figure 4B).

**Figure 5.**
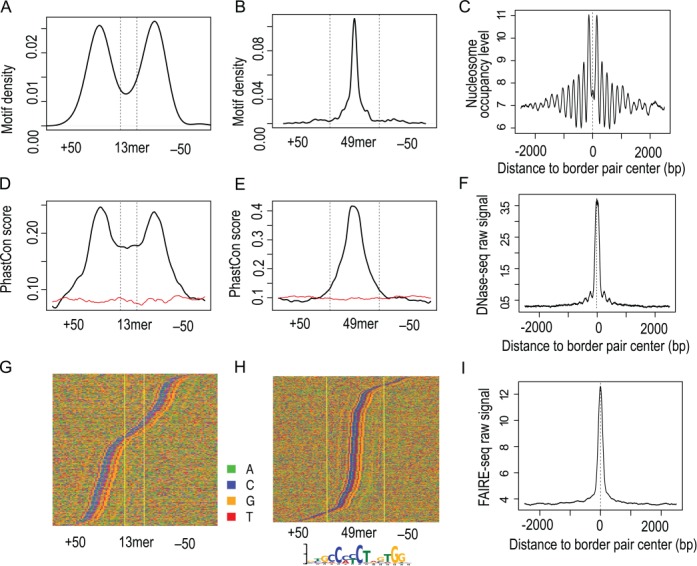
Evaluate MACE-identified CTCF binding sites using motif, conservation, nucleosome position and DNA accessibility. The 13mer peaks identified by Rhee *etal*. (2011) were also included for comparison purpose. Column 1 (panels A, D and G), evaluation of the 13mer peaks identified by Rhee *etal*. using CTCF motif density, conservation and direct sequence pileup. Column 2 (panels B, E and H) shows evaluation of 49mer border pairs identified from MACE using CTCF motif density, conservation and direct sequence pileup. Column 3 (panels C, F and I), evaluation of all border pairs identified by MACE MNase-seq tag density, DNase-seq tag density and FAIRE-seq tag density. Conservation was measured by PhastCon score calculated from multiple alignments of 46 vertebrate genomes. Nucleotides are illustrated with different colors: A (green), C (blue), G (orange) and T (red). We extended 50 nt upstream and downstream of border pairs.

We next evaluated identified CTCF border pairs. We selected 13mer and 49mer border pairs and checked the motif distribution, sequence conservation and direct border-pair DNA sequence pileups. As expected from Figure 4, we found that for 49mer border pairs, both motif density and conservation score profile showed a single peak centered on the border pairs, indicating that real CTCF bindings were encompassed perfectly (Figure 5B, E and H). However, the bimodal distributions of motif density and conservation that we observed for 13mer border pairs suggested that these border pairs were located on either side of the motif, rather than centered on it. (Figure 5A, D and G, Supplementary Figure S4B).

One key function of CTCF is to regulate chromatin structure, and its binding sites have been used to position nucleosomes (32). We first aligned peak pairs by the midpoint, then extended by 2 kb upstream and downstream, and measured the nucleosome occupancy level using published MNase-seq data (17). We found that CTCF border pairs were located in NFRs and could position 20 or more nucleosomes around them (Figure 5C). The authenticity of these identified binding sites were further verified by both DNase-seq and FAIRE-seq data generated by the ENCODE consortium from the same cell type (Figure 5F and I).

Using the same MNase-seq dataset, we performed similar analyses for peaks identified from ENCODE CTCF ChIP-seq and peaks identified from CTCF ChIP-exo by Rhee *et al.* In general, we found much weaker nucleosome oscillation patterns and fewer well-positioned flanking nucleosomes (Supplementary Figure S9A). Then we asked if MACE identified border pairs without CTCF motifs could be used as anchor points to position a nucleosome. Since whether a motif can be aligned to DNA sequences depends on the number of mismatches allowed, we divided all border pairs into six groups according to the edit distance between DNA sequence and the CTCF motif (i.e. 0-mismathc, 1-mismatch, 2-mismatch, 3-mismatch, 4-mismatch, 5-or-more mismatches). We defined border pairs having ‘5-or-more mismatches’ with canonical CTCF motif as those ‘without CTCF motif’ because 66% of sequences randomly selected from the genome would have CTCF motif if ‘5-or-more mismatches’ were allowed (Supplementary Figure S10). As shown in Supplementary Figure S9B, MACE border pairs without CTCF motif were still able to position 20+ nucleosomes precisely, and the authenticity of these border pairs were confirmed by DNaseI and FAIRE-seq signals (Supplementary Figure S9C and D). This demonstrated both the enhanced accuracy of ChIP-exo and the power of MACE.

### Identification of HNF6 binding sites in the mouse genome

**Figure 6.**
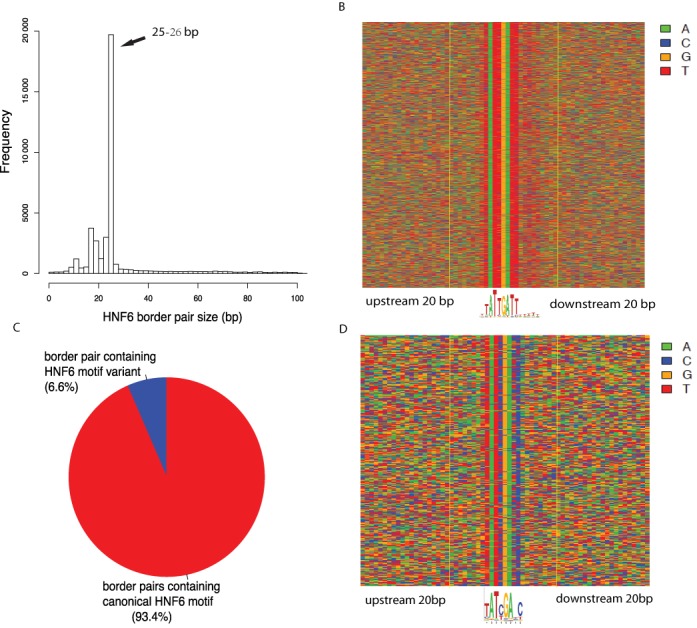
(**A**) Size distribution of HNF6 border pairs. (**B**) Direct sequence pileup of 25mer border pairs. (**C**) Pie chart showing the frequency of canonical motif and motif variant. (**D**) Direct sequence pileup of border pairs containing HNF6 motif variant. Nucleotides were illustrated with different colors: A (green), C (blue), G (orange) and T (red). Border pairs were defined by two vertical yellow lines.

We applied MACE to our own mouse ONECUT1 ChIP-exo data (Supplementary Table S3). Out of the identified border pairs, 25mer is the most abundant one, suggesting the *in vivo* binding size of HNF6 was 25 bp. (Figure 6A). We then extracted genome sequences of those 25mer border pairs and piled them up directly. The motif (TATTGATT) was visually recognizable and located right in the middle of the border (Figure 6B). This motif corresponds to the recently reported human liver ONECUT1 ChIP-seq (33). In addition, the downstream polyT tract within 25mer border pair was also identifiable by inspection, even though the signal was weaker. It was recently reported that traditional motif representation methods relying upon residue frequencies couldn't effectively visualize weak signals (34). Using an algorithm implemented in plogo, we found the downstream polyT was significantly enriched, which accords with the ONECUT motif recently obtained using HT-Selex that was predicted to narrow the minor groove of DNA (Supplementary Figure S11) (35). If we focused on those border pairs with high confidence (i.e. both borders were well defined and the border pair size is 25 bp), we found 93.4% of them contained canonical ONECUT1 (Figure 6C). We identified an ONECUT1 motif variant (TATYGANC) by directly piling up the remaining 6.6% border pairs (Figure 6D).

In summary, we found MACE could demarcate HNF6 binding boundaries in mouse genomes with high accuracy, and precisely defined border pairs were useful to improve motif detection, especially weak residues. We also demonstrated that ChIP-exo data is useful to identify low abundance motif variant, which might be overlooked by traditional motif detection methods because of the dominance of the canonical motif.

### Direct comparison reveals the superior performance of MACE

**Figure 7.**
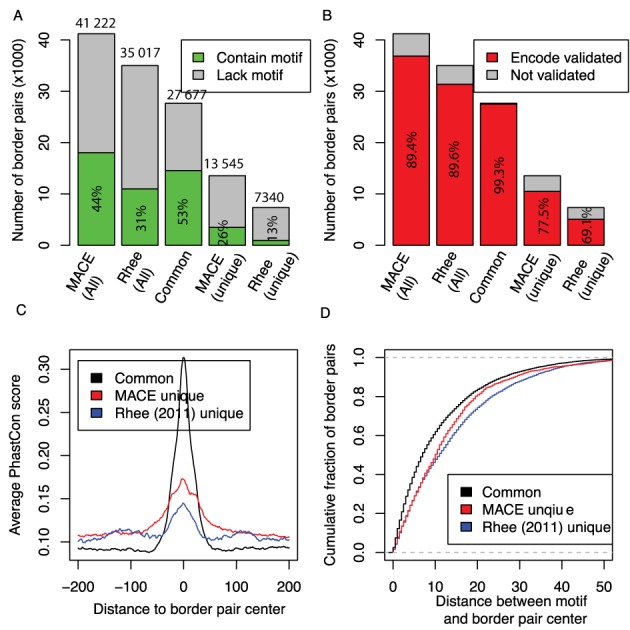
Comparing MACE with the method used by Rhee *etal*. (2011). (**A**) Bar-plot showing percent of putative binding regions (green areas) supported by CTCF canonical motif (RSYDMCMYCTRSTGK). The bar height indicates the number of total binding regions (exact numbers are superimposed on top). (B) Bar-plot showing percent of putative binding regions (red area) cross-validated by ENCODE CTCF ChIP-seq. (**C**) Comparing average conservation score (PhastCon) between regions identified by MACE and Rhee 2011. All binding regions identified by the two methods were combined and then divided into three groups: common border pair (black), MACE-unique (red) and Rhee *etal*.-unique (blue). (**D**) Cumulative curves used to compare spatial resolution between MACE and Rhee 2011. CTCF indicates CCCTC binding factor; MACE, model-based analysis of ChIP-exo.

Using a pseudo *P*-value cutoff of 0.05, we identified 41 222 unique CTCF border pairs (52 084 with redundancy), of which 18 023 (43.72%) had canonical CTCF motif (RSYDMCMYCTRSTGK) (Supplementary Table S2). To estimate the enrichment of CTCF motifs, we randomly shuffled genome coordinates of these border pairs, and found only 977 (2.37%) had the CTCF motif. In other words, the CTCF motif was enriched 18.45-fold in MACE identified border pairs, compared to background. As a comparison, we performed the same calculation for binding sites identified by Rhee *et* *al*.: 10 970 of 35 017 regions (31.33%) encompassed the CTCF motif. MACE identified 6205 putative regions not reported in the original paper, suggesting greater sensitivity. More importantly, a higher proportion of border pairs detected by MACE had a supporting CTCF motif (43.72 versus 31.33% in the original paper), suggesting higher specificity (Figure 7A). Additionally, we combined binding regions identified from MACE and Rhee *et* *al*. together and divided them into three groups: Common (27 677), MACE-unique (13 545) and Rhee 2011-unique (7340). The CTCF motif was encompassed by 52.6% (14 553) of common binding regions, 25.6% (3470) of MACE-unique regions and 12.9% (946) of Rhee 2011-unique regions (Figure 7A).

We also used ENCODE CTCF ChIP-seq data generated from the same HeLa cell line as an independent evidence to compare MACE with the Rhee 2011 peak calling method. Overall 89.4% (36 867) of putative regions identified by MACE and 89.6% (31 374) identified by Rhee 2011 method were concordant with ChIP-seq results (Figure 7B). We divided all putative regions into three groups as above and found that that 99.3% (27 495) of common binding sites, 77.5% (10 503) of MACE-unique sites and 69.1% (5070) of Rhee 2011-unique binding sites were verified with ENCODE ChIP-seq results. Consistent with the MACE-unique group having more genuine binding sites than that of the Rhee 2011-unique group, we also found that the binding regions in the MACE-unique group were more evolutionary conserved (Figure 7C). Finally, when measuring the spatial resolution by the distance between the center of CTCF motif and the center of putative binding regions, we observed that MACE had better resolution than the method used by Rhee *et* *al*. (Figure 7D).

Two computational tools Genome Positioning System (GPS) (15) and genome-wide event finding and motif discovery (GEM) (16) were developed and successfully applied to TFBS prediction using ChIP-exo data. For technical reasons, we were unable to perform a fair comparison between MACE and GEM (see ‘Discussion’ section). Based on cross strand correlation analysis, we estimated the size of DNA fragment is 50 bp. Therefore, we extended the GPS reported binding centers in both directions by 25 bp as the GPS detected binding events. We then searched canonical CTCF motif in these two lists of TFBSs, 43.7% (42.5%) of MACE (GPS) detected TFBSs encompassing a CTCF motif (Supplementary Figure S12A). When evaluated using ENCODE ChIP-seq results, 89.4% (88.7%) of MACE (GPS) identified TFBSs could be verified (Supplementary Figure S12B). We measured the spatial resolution by distance between TFBS center and CTCF motif center; the median resolutions for MACE and GPS were 4.5 and 7 nt, respectively (Supplementary Figure S12C and D). Overall, these results indicated that MACE has better motif coverage, higher spatial resolution (*P*-value < 2.2e-16, Wilcoxon rank sum test) and higher validate rate than GPS.

**Figure 8.**
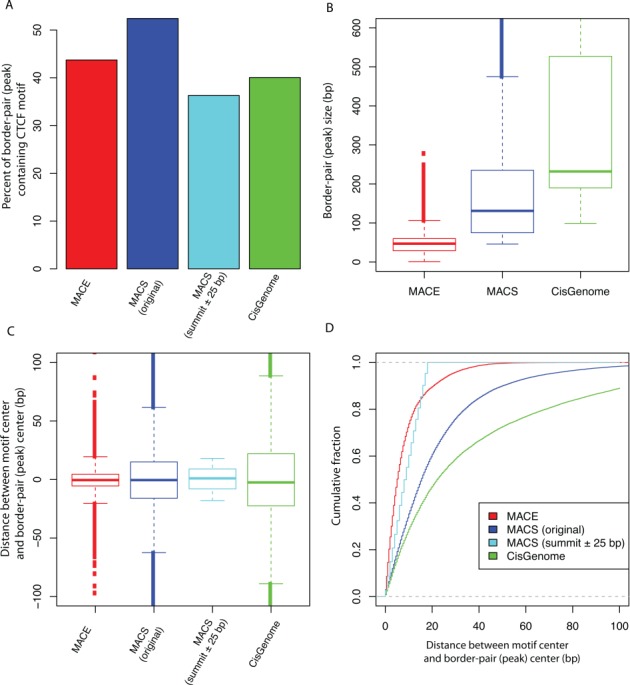
Comparing MACE with ChIP-seq peak calling tools (MACS and CisGenome). (**A**) Motif enrichment in peaks or border pairs. Enrichment is measured by percentage of peaks or border pairs encompassing motif. (**B**) Peak (border pair) size. (**C**) and (**D**) Spatial resolution measured by distance between motif center and midpoint of peaks (border pairs). ‘MACS (original)’ refers to peaks originally reported by MACS. ‘MACS (summit ± 25 bp)’ refers to peaks that only keep the 51 bp centered on summit (i.e. extending 25 bp up- and downstream to peak-summit).

Many tools have developed to analyze ChIP-seq data. Here we compared MACE to MACS (v2.0.10) and CisGenome (v1–1.2) using the same CTCF ChIP-exo data (7,10). All three software were running with their default configurations. 43, 52 and 40% of MACE, MACS and CisGenome detected peaks contained canonical CTCF motif with MACS had the highest motif enrichment (Figure 8A). However, motif enrichment was also affected by the peak (or border pair) size. As shown in Figure 8B, the peak sizes of MACS (median = 131 bp) and CisGenome (median = 232 bp) were much larger than that of MACE (median = 47 bp). We then measured the resolution using the distance between motif and the center of peaks (or border pairs). MACE (median = 4.5 bp) achieved the best resolution compared to MACS (median = 15.5) and CisGenome (median = 22.5 bp) (Figure 8C). And 90% of motifs in MACE border pairs were located within 20 bp around the midpoint, while only 60 and 47% of motifs in MACS and CisGenome detected peaks were located within 20 bp around the midpoints (Figure 8D). We also prepared another version of MACS peaks [referred as MACS (summit ± 25 bp) in Figure 8] by extending 25 bp to peak summit to both up- and downstream. Therefore, this list of peaks has constant size of 51 bp, and always centers on the summit. As shown in Figure 8A, only 36% of MACS (summit ± 25 bp) peaks contained CTCF motif because of their relative smaller size compared to original MACS peaks. We found MACE border pairs still have better spatial resolution than MACS (summit ± 25 bp) and MACS (original) peaks with the median distance for MACE, MACS (original) and MACS (summit ± 25 bp) are 4.5, 15.5 and 9 bp, respectively. These results suggested that although ChIP-seq peak-calling algorithms can be applied to ChIP-exo data, these tools might not take full advantages (i.e. high resolution) of ChIP-exo data.

## SUMMARY AND DISCUSSION

ChIP-exo is technically different from ChIP-seq, and therefore data generated from these two platforms should be processed differently. First, although biological replicates are generally required in ChIP-seq experiments, it has been reported that more than two replicates did not significantly improve site discovery (14,36). This is because ChIP sequencing can be easily saturated and additional replicates in ChIP-seq are mainly used as a validation set. However, replicates in ChIP-exo are used to reduce spurious ‘noisy borders’ and enhance spatial resolution. Using CTCF ChIP-exo data, we showed that spurious signals could be reduced much greater using three replicates than using two replicates (Supplementary Figure S13), suggesting multiple replicates and noise reduction are critical for precise border definition. We exemplified in this study that relative entropy combined ‘replicate consolidation’ and ‘noise reduction’ in a single step and outperformed other methods. This approach is also potentially useful for other high-throughput sequencing-based data (such as RNA-seq) analysis. Second, based on the design philosophy of ChIP-exo, MACE expects over-represented signals at genomic positions where λ exonuclease stopped during DNA digesting, and these positions are potential protein-binding boundaries *in vivo*. This distinguishes MACE from most peak calling algorithms designed for ChIP-seq (in ChIP-seq, such overrepresented signals are generally considered as PCR over-amplification bias and should be recalibrated properly). Computational tools such as GPS and GEM have been developed and were successfully used to predict TFBS using ChIP-exo data, but both tools were not designed to take advantage of this unique feature (i.e. overrepresented signals signify binding borders) to improve resolution (15,16). Because of the assumption that over-represented signals were expected at certain genomics positions for a particular TFBS, MACE cannot be applied to traditional ChIP-seq data.

TFs play key roles in establishing nucleosome positioning and many studies have reported an association between TFBS and nucleosome positions (4,30,32,37). However, the exact relationship between TFBS and nucleosome positioning is not yet fully understood due to the lack of large-scale, high-resolution experimental data. Our results indicated that Reb1 binding in the yeast genome could position 20 nucleosomes within 3 kb regions (Figure 3D and E) and that CTCF binding in the human genome could position 20 or more nucleosomes within a 4-kb region (Figure 5C). Note that no motif information was used to increase spatial resolution during binding-site detection; which highlights higher resolution of ChIP-exo and the advantages of MACE.

When evaluating Reb1 border pairs, we found that 94% of border pairs (1118 of 1194) identified by MACE encompassed the Reb1 motif, demonstrating high specificity. When comparing MACE with the method used by Rhee *et* *al*. (4), we reported that 50% or fewer of the putative border pairs encompassed the CTCF motif (Figure 7A); this was because we used only the canonical CTCF motif retrieved from the JASPAR database. Considering that the CTCF protein has up to six degenerate motifs, we expected that the percentage of CTCF border pairs having a CTCF motif would be considerably higher. We searched the 15mer canonical CTCF motif in candidate border pairs with mismatches up to five. When allowing four mismatches, 72% of detected border-pairs encompassed CTCF motifs (Supplementary Figure S10). The border pairs containing motifs with a four-nucleotide difference from the canonical one were still likely to be the real binding, because of the enriched CTCF motif (Supplementary Figure S10), similar tag intensity profile (Supplementary Figure S14E) and higher sequence conservation compared to genome background (Supplementary Figure S14F).

During the development of MACE, GEM was developed and applied to ChIP-exo data analyses (15,16). GEM achieves its high spatial resolution by reciprocally improving motif detection, using binding event locations and binding event predictions made with discovered motifs. It is difficult to compare MACE and GEM because the DNA motif is the only independent evidence that can be used to evaluate spatial resolution, and the motif is already used by GEM to improve resolution, although some TFBSs are reported without the DNA motif. In addition, the focus of GEM and MACE are different in that GEM reports single-nucleotide positions, whereas MACE reports regions (border pairs). For example, with MACE, we can estimate that the border pair size of Reb1, HNF6 and CTCF is 27, 25 and 49 bp respectively.

## AVAILABILITY

Raw and processed data of CTCF ChIP-exo, source code and documentation of MACE are available at http://chipexo.sourceforge.net/. Raw ONECUT1 ChIP-exo data was deposited in Array Express with accession number E-MTAB-2060.

## SUPPLEMENTARY DATA

Supplementary Data are available at NAR Online.

SUPPLEMENTARY DATA
