# Supplementary material for: MACE: model based analysis of ChIP-exo
Source: Nucleic Acids Res. 2014 Sep 23;42(20):e156. doi: 10.1093/nar/gku846 (PMC4227761; doi:10.1093/nar/gku846)
Supplement: SUPPLEMENTARY DATA [file supp_42_20_e156__index.html]

MACE: model based analysis of ChIP-exo — MACE: model based analysis of ChIP-exo — SUPPLEMENTARY DATA 

# MACE: model based analysis of ChIP-exo

## SUPPLEMENTARY DATA

**Files in this Data Supplement:**

- SUPPLEMENTARY DATA
- SUPPLEMENTARY DATA
