# Supplementary material for: MACE: model based analysis of ChIP-exo
Source: Nucleic Acids Res. 2014 Sep 23;42(20):e156. doi: 10.1093/nar/gku846 (PMC4227761; doi:10.1093/nar/gku846)

Supplementary Materials for

MACE: Model based analysis of ChIP-exo

Liguo Wang[1,4*], Junsheng Chen[3], Chen Wang[1], Liis Uusküla-Reimand[5], Kaifu Chen[4], Alejandra Medina-Rivera[5], Edwin J. Young[5], Michael T. Zimmermann[1], Huihuang Yan[1], Zhifu Sun[1], Yuji Zhang[1], Stephen T. Wu[1], Haojie Huang[2], Michael D. Wilson[5,6], Jean-Pierre A. Kocher[1*], Wei Li[4*]

[1]Division of Biomedical Statistics and Informatics, Mayo Clinic, Rochester, MN 55905, USA;
[2]Department of Biochemistry and Molecular Biology, Mayo Clinic, MN 55905, USA;
[3]School of Life Science and Technology, Tongji University, Shanghai 200092, China.
[4]Division of Biostatistics, Dan L. Duncan Cancer Center and Department of Molecular and Cellular Biology, Baylor College of Medicine, Houston, TX 77030, USA
[5]Genetics & Genome Biology Program, SickKids Research Institute, 686 Bay Street. Toronto, ON, M5G 0A4, Canada
[6]Department of Molecular Genetics, University of Toronto, Canada

* To whom correspondence should be addressed:

Tel: +1 507 284 8728
Fax: +1-507-284-0360
Email: wang.liguo@mayo.edu

Tel: +1 507 538 8315
Fax: +1 507 284 0360
Email: kocher.jeanpierre@mayo.edu,

Tel: +1 713 798 7854
Fax: +1 713 798 6822
Email: wl1@bcm.edu

Running title: Demarcation of Protein-DNA Binding Boundaries

This PDF file includes:

Supplementary Figure 1
Supplementary Figure 2
Supplementary Figure 3
Supplementary Figure 4
Supplementary Figure 5
Supplementary Figure 6
Supplementary Figure 7
Supplementary Figure 8
Supplementary Figure 9
Supplementary Figure 10
Supplementary Figure 11
Supplementary Figure 12
Supplementary Figure 13
Supplementary Figure 14
Supplementary Figure 15

Supplementary Figure legends

Supplementary Figure 1
Nucleotide composition (y-axis) bias in multiple ChIP-exo datasets. A, Mouse ONECUT1 (HNF6). B, Yeast Reb1. C, Human CTCF. D, Human CTCF with bias corrected.

Supplementary Figure 2
Effects of nucleotide composition bias correction illustrated by the coverage profile around CTCF motifs. Vertical dashed curves indicate CTCF motif position.

Supplementary Figure 3
Evaluating the impact of “nucleotide composition bias correction” and “entropy-based noise reduction” upon border-pair detection. A, Validate detected border pairs using ChIP-seq results from ENCODE. B, Validate detected border pairs using CTCF motif. C, Spatial resolution measured by distance to motif.

Supplementary Figure 4
Screenshot from the University of California, Santa Cruz genome browser. Twelve custom tracks are displayed. From top to bottom: coverage profiles from 3 biologic replicates, calculated from reads mapped to the forward strand (dark blue); coverage profiles from 3 biologic replicates, calculated from reads mapped to the reverse strand (dark red); signal consolidated from the 3 forward-strand reads (dark blue); signal consolidated from the 4 reverse-strand reads (dark red); border pairs called by MACE (blue); peaks detected by Rhee 2011 (green); in silico predicted CTCF motif (red); phastCon conservation score in mammals (dark green). A, CTCF binding site on

promoter region of Myc. B, Example showing peak identified by Rhee et al, 2011 was off target.

Supplementary Figure 5
Relationship between entropy-based noise reduction effects and signal intensity. All predicted CTCF motifs were ranked by ChIP-exo tag intensity in descending order and equally divided into 4 groups: A, the first quantile (0-25%) represented the strongest bindings; B, the second quantile (25-50%) represented modest strong bindings; C, the third quantile (50-75%) represented modest weak bindings; D, the fourth quantile (75-100%) represented weakest binding. Two vertical dashed lines indicate the CTCF motif position.

Supplementary Figure 6
A, Reb1 border pair size distribution. B, Reb1 motif density profile over 26mer border pairs. C, Conservation profile over 26mer border pairs. D, Direct sequence pileup of 26mer border pairs.

Supplementary Figure 7
Genomic distribution of Reb1 border pairs encompassing motif (blue) and background control (black). TSS indicates transcription start sites.

Supplementary Figure 8
ChIP-exo raw sequencing tags profile over Reb1 motifs. Blue represent forward tags and red represent reverse tags.

Supplementary Figure 9
A, MNase-seq tag intensity profiles around peaks detected by ENCODE (red) and Rhee et al. (blue) B, MNase-seq tag intensity profiles around MACE detected border pairs. Border pairs were stratified into 6 groups (0-mismath, 1-mismath, 2-mismath, 3-mismath, 4-mismath and 5-or-more mismatches) according to its editing distances to canonical CTCF motif. C, DNaseI-seq intensity profiles around 6 groups of MACE border pairs. D, FAIRE-seq intensity profiles around 6 groups of MACE border pairs.

Supplementary Figure 10
Comparison of CTCF motif enrichment in binding regions defined by MACE (red), Rhee et al (blue) and genome background (black). X-axis indicated "number of mismatches" allowed when searching CTCF motif in candidate binding regions.

Supplementary Figure 11
ONECUT1 (HNF6) motif identified from 25mer border pairs. Motif logo was generated using plogo (http://plogo.uconn.edu/).

Supplementary Figure 12
Performance comparison between MACE and GPS. A, Bar-plot showing percent of putative binding regions (red areas) supported by CTCF canonical motif (RSYDMCMYCTRSTGK). B, Bar-plot showing percent of putative binding regions

validated by ENCODE CTCF ChIP-seq. C and D, Compare spatial resolution between MACE and GPS. Spatial resolution was measured by distance between motif and peak center (y-axis in C and x-aixs in D).

Supplementary Figure 13
Entropy based noise reduction effects using 2 replicate (dashed curves) and 3 replicates (solid curves). Forward and reverse strand signals were represented in blue and red, respectively. All predicted CTCF motifs were ranked by ChIP-exo tag intensity in descending order and equally divided into 4 groups: A, the first quantile (0-25%) represented the strongest binding; B, the second quantile (25-50%) represented modest strong binding; C, the third quantile (50-75%) represented modest weak binding; D, the fourth quantile (75-100%) represented weakest binding. Rep123, using all 3 replicates; Rep12, using replicate-1 and replicate-2; Rep13, using replicate-1 and replicate-3; Rep23, using replicate-2 and replicate-3. Two vertical dashed lines indicate the CTCF motif position.

Supplementary Figure 14
A, Tag intensity profile of border pair with exact (0-mismatch) CTCF motif. B, C, D, E Tag intensity profiles of border pair with 1-, 2-, 3- and 4-mismatch to canonical CTCF motif, respectively. F, Sequence conservation profiles of border pair with 0- (black), 1- (red), 2- (green), 3- (blue) and 4- (cyan) mismatch to canonical CTCF motif. Dashed line indicated genome background.

Supplementary Figure 15
Comparing signal-to-noise ratio between ChIP-seq and ChIP-exo. We sampled the same number of reads (i.e. 10 million) from both ChIP-seq and ChIP-exo experiments, and trimmed reads to the same length. Blue dots represented binding sites detected by both ChIP-seq and ChIP-exo. X-axis: tag intensity (measured by wigsum) of ChIP-exo; Y-axis: tag intensity of ChIP-seq. Red dashed curve indicated diagonal line (slope = 1) and red solid curve indicated regression line.

Supplementary Figure 1

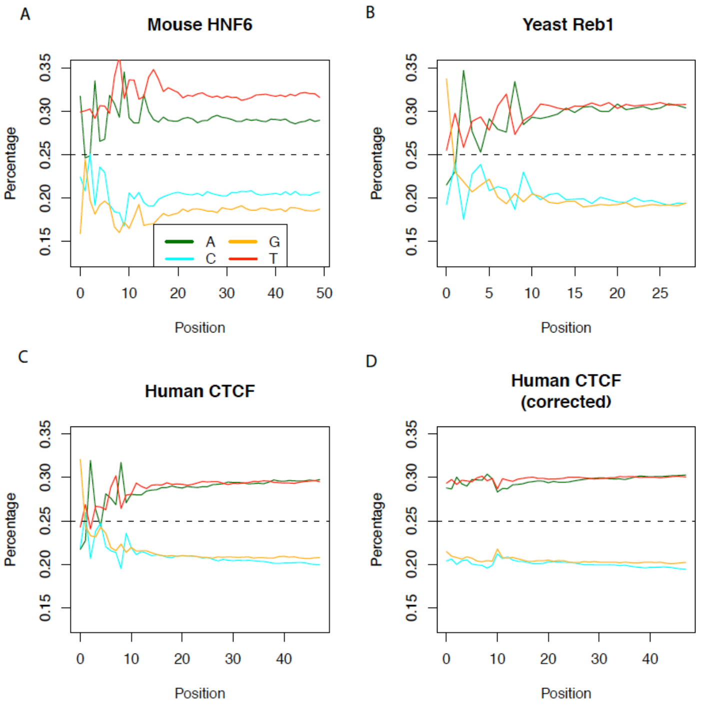

Supplementary Figure 2

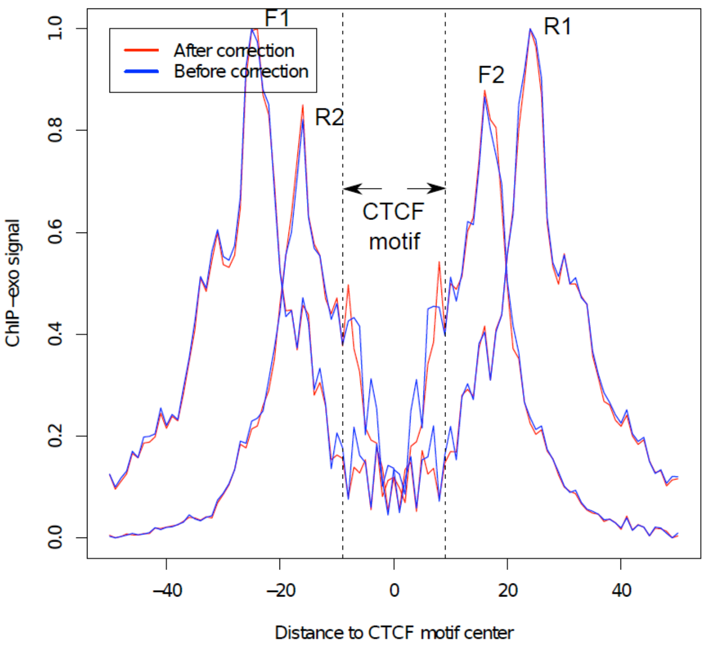

Supplementary Figure 3

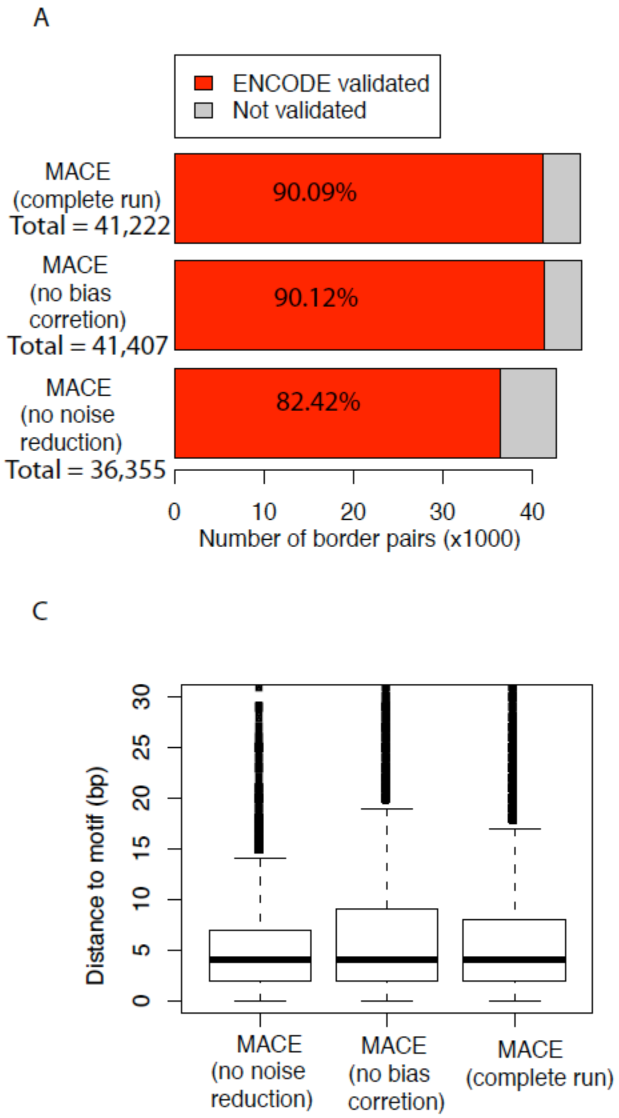

Supplementary Figure 4

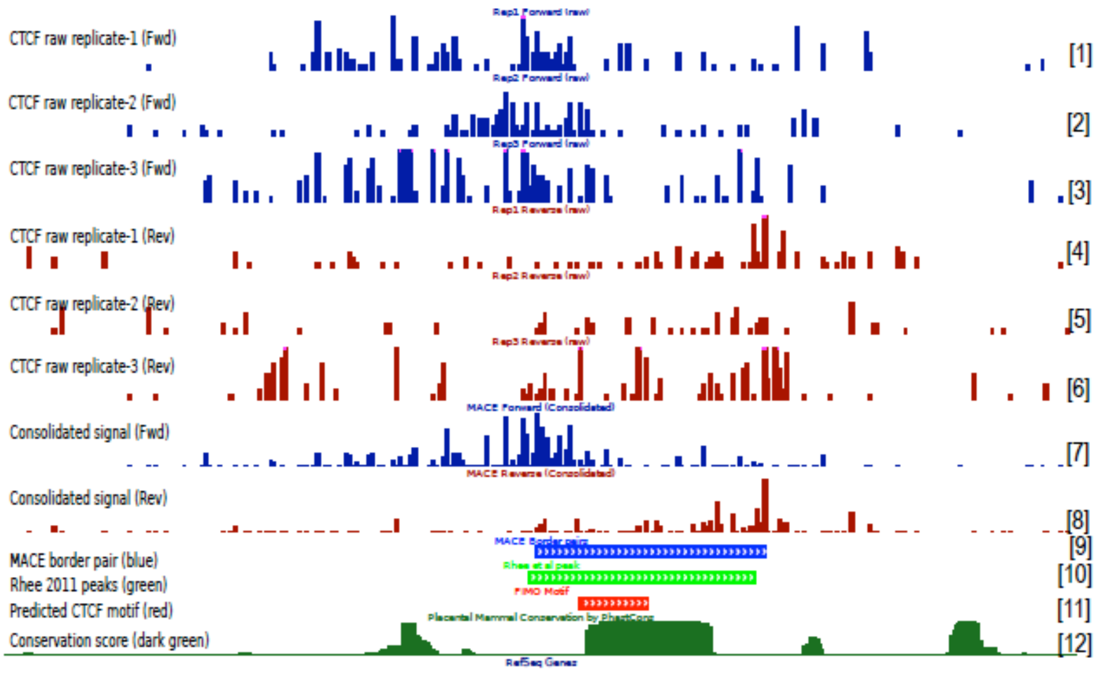

B

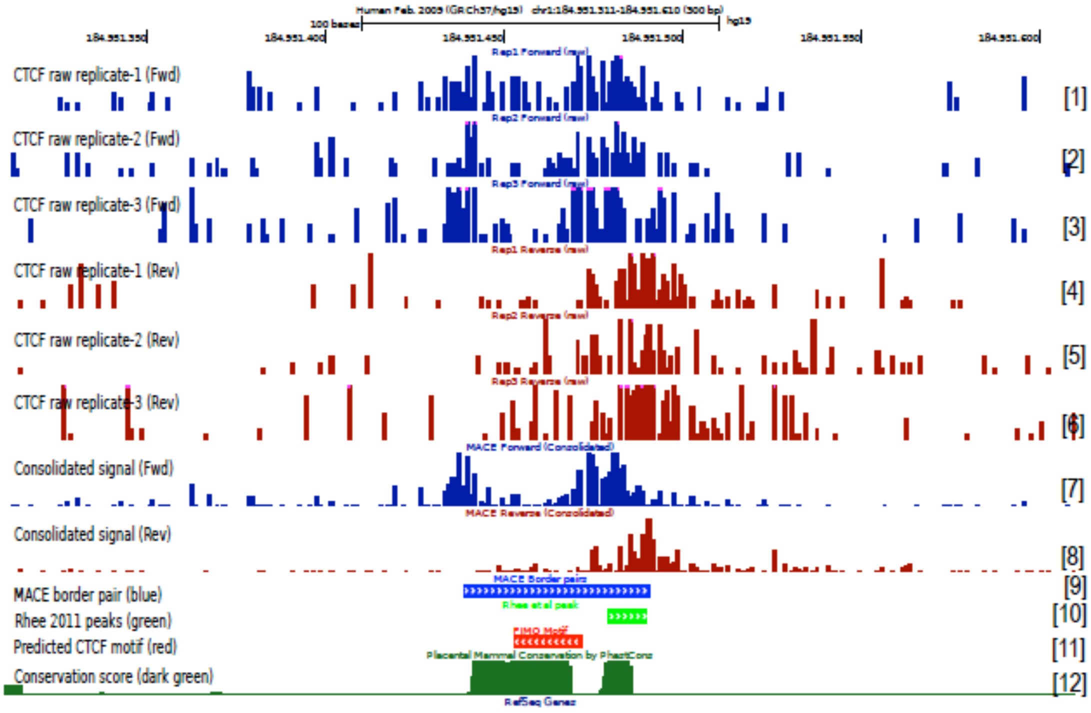

Supplementary Figure 5

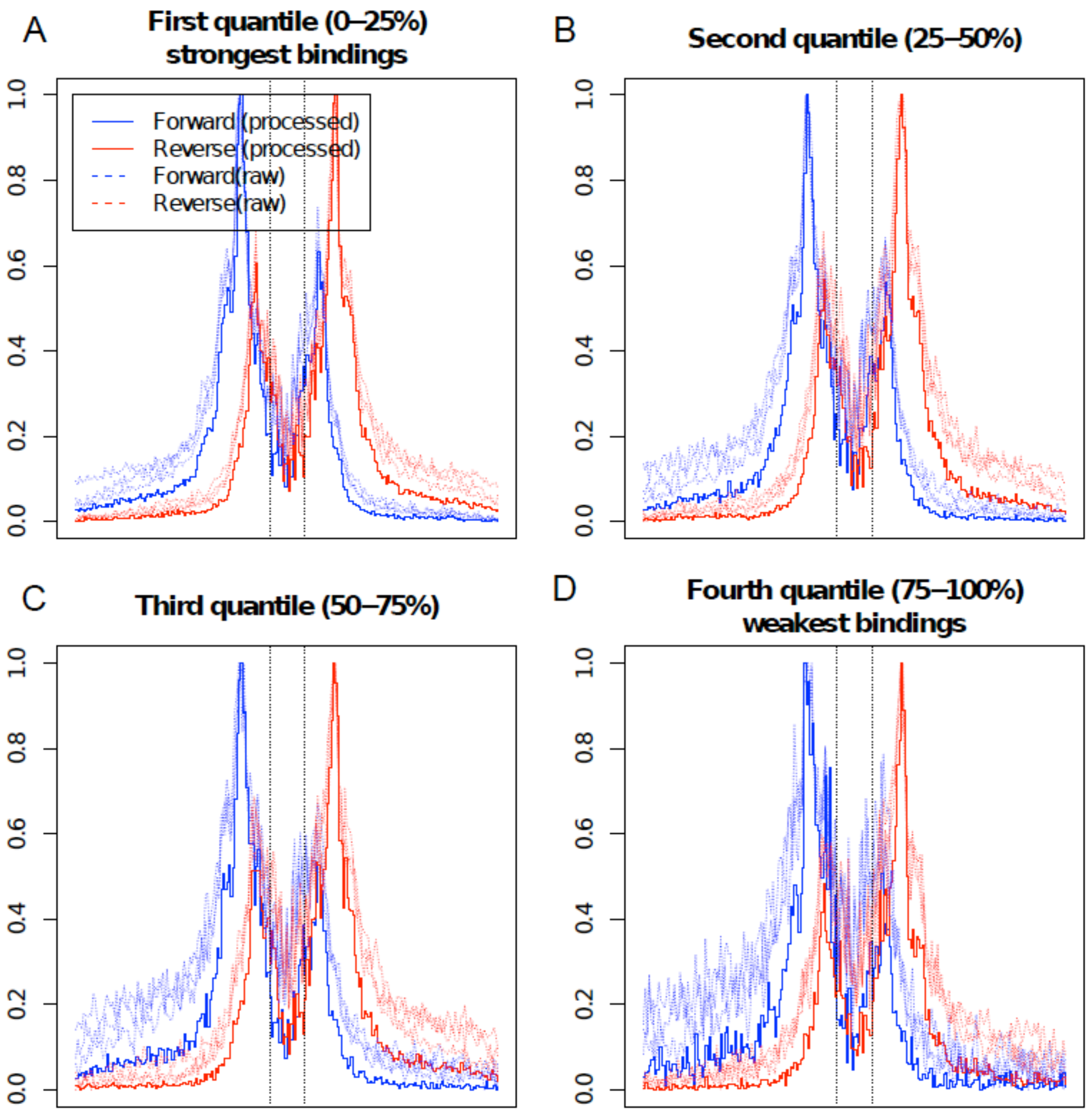

Supplementary Figure 6

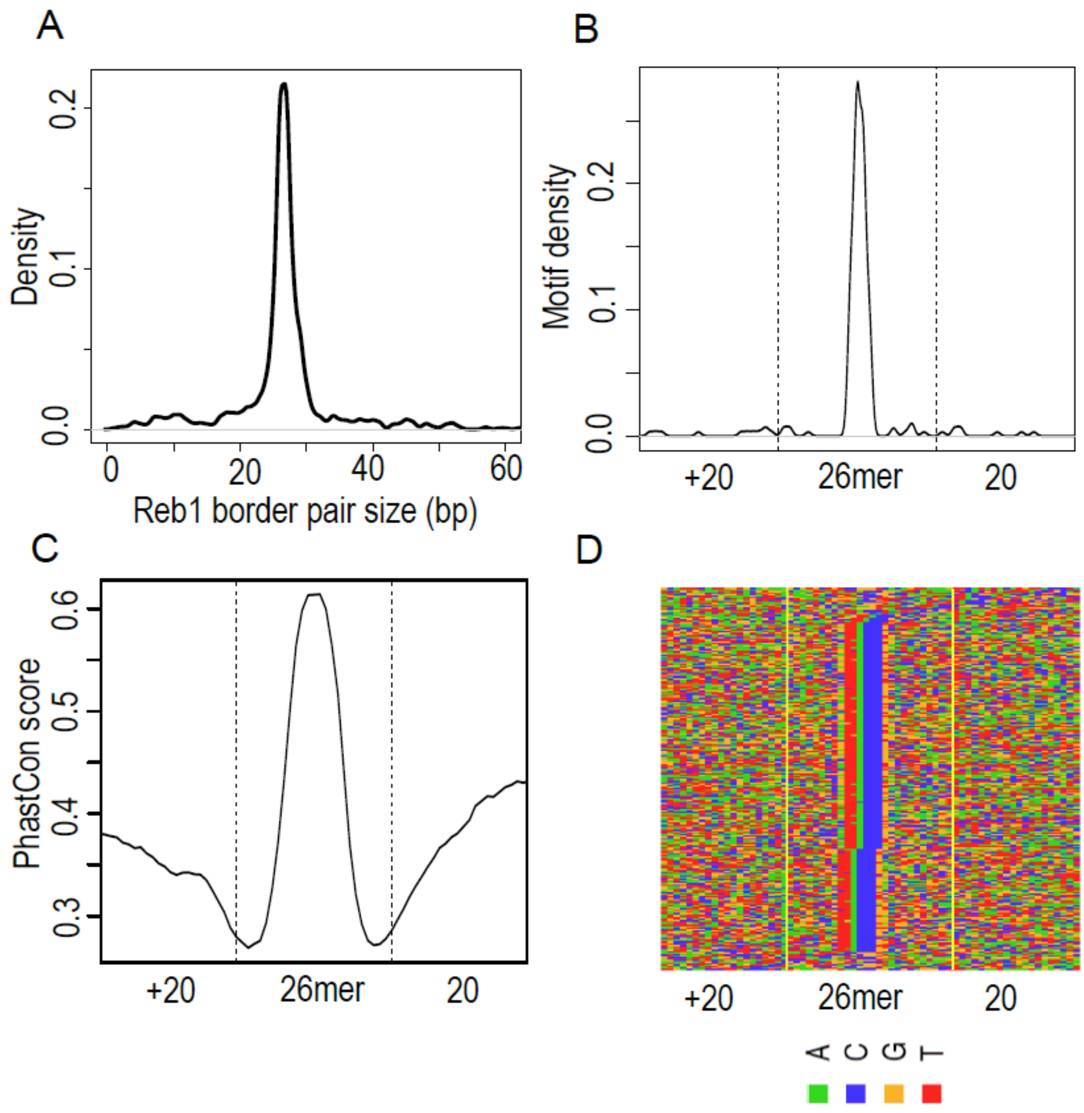

Supplementary Figure 7

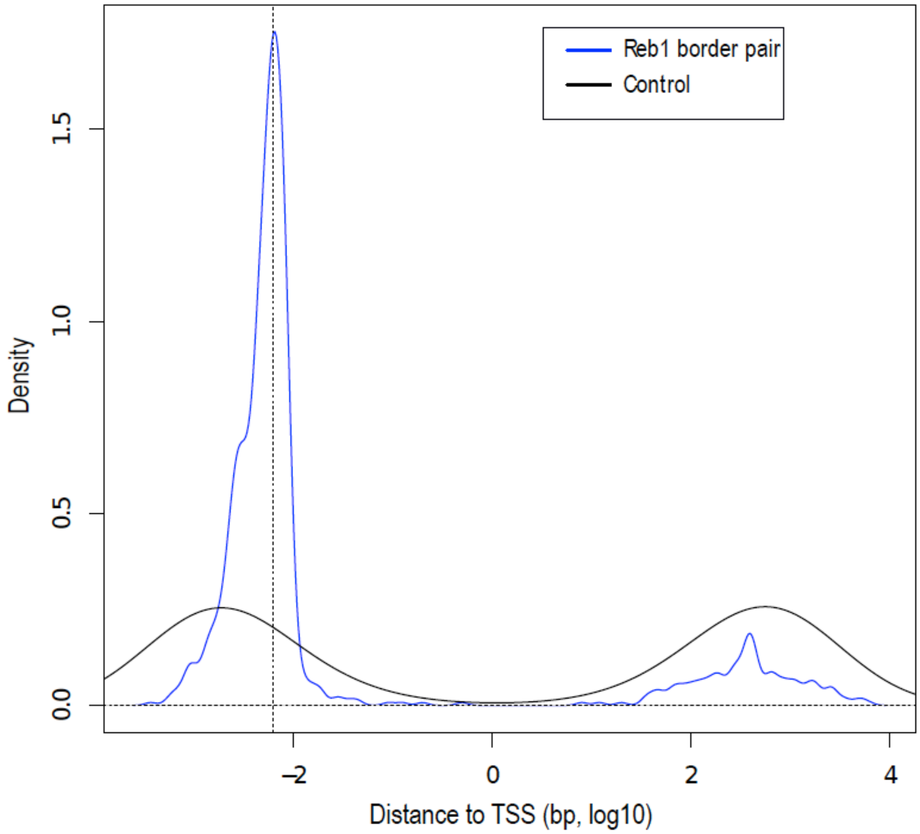

Supplementary Figure 8

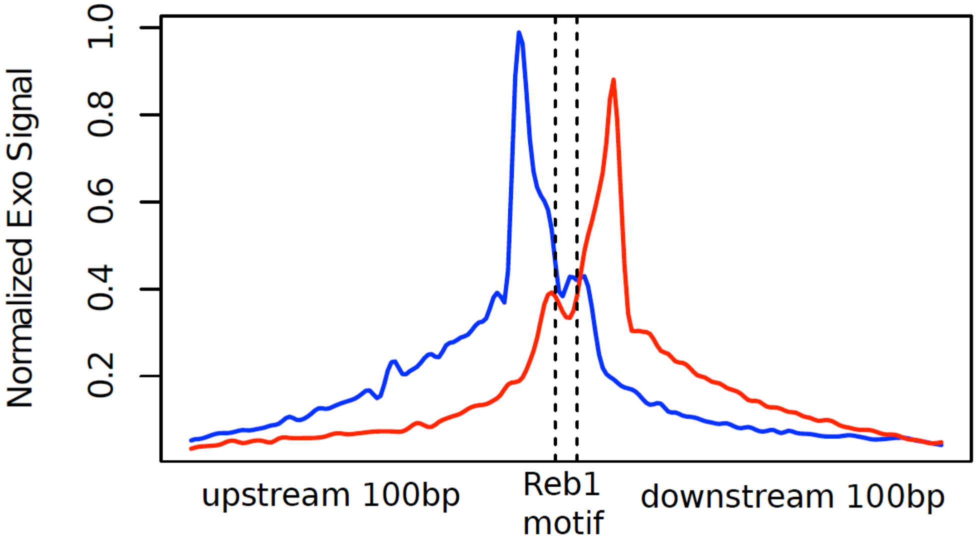

Supplementary Figure 9

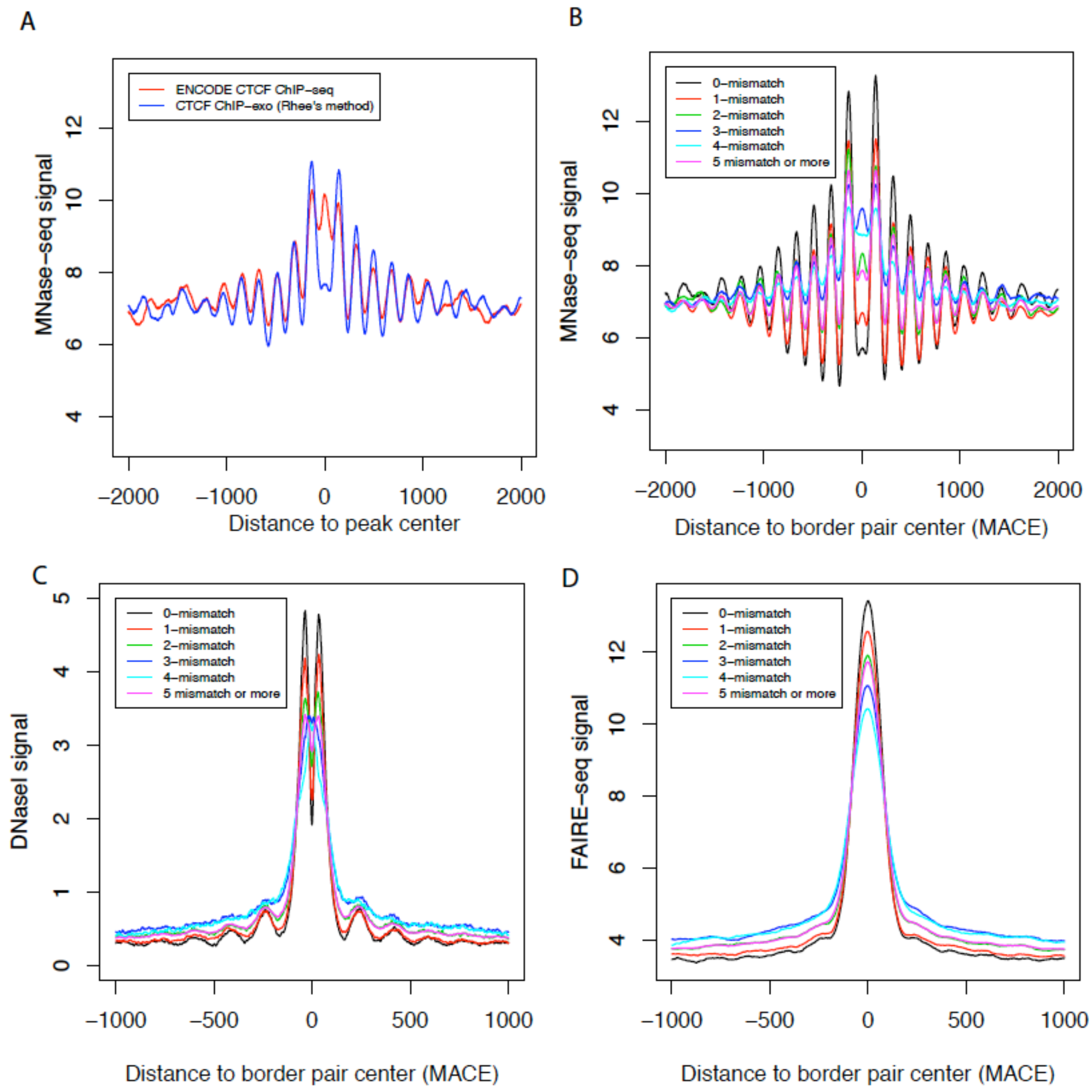

Supplementary Figure 10

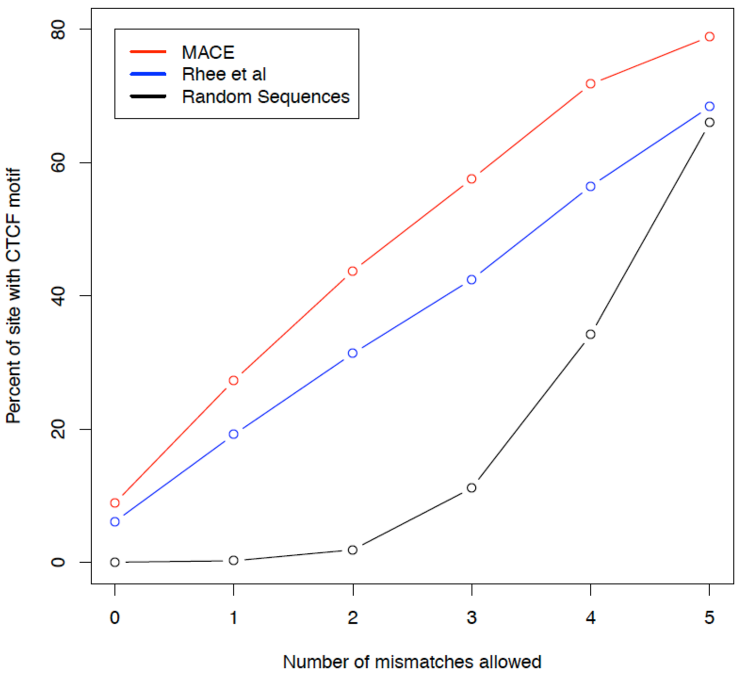

Supplementary Figure 11

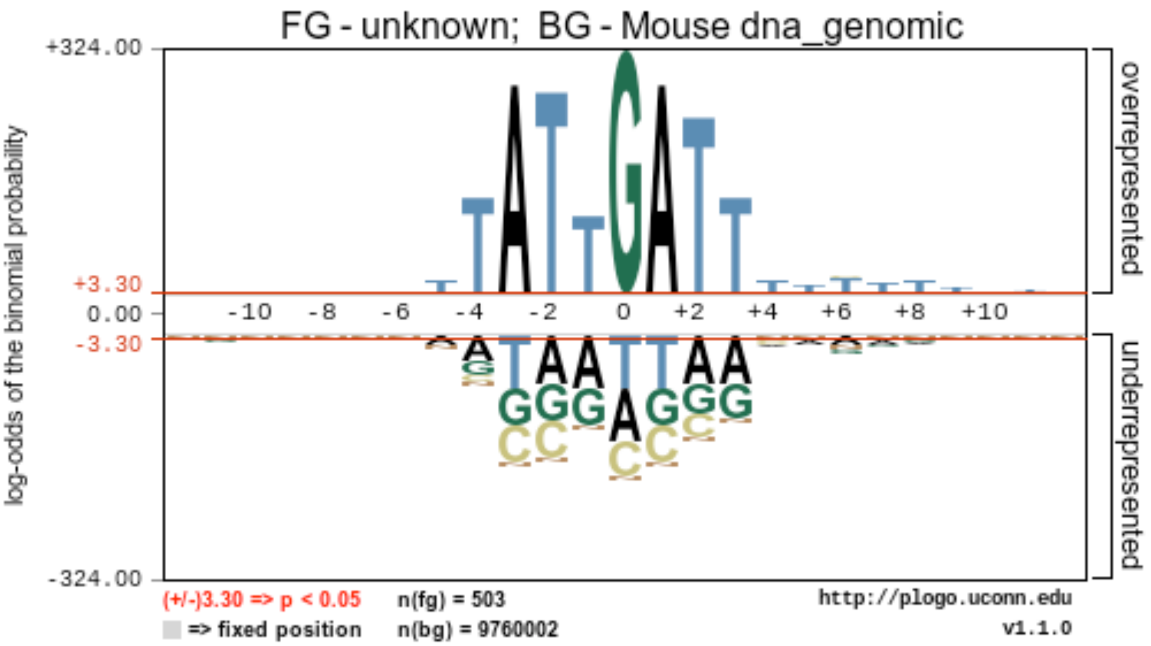

Supplementary Figure 12

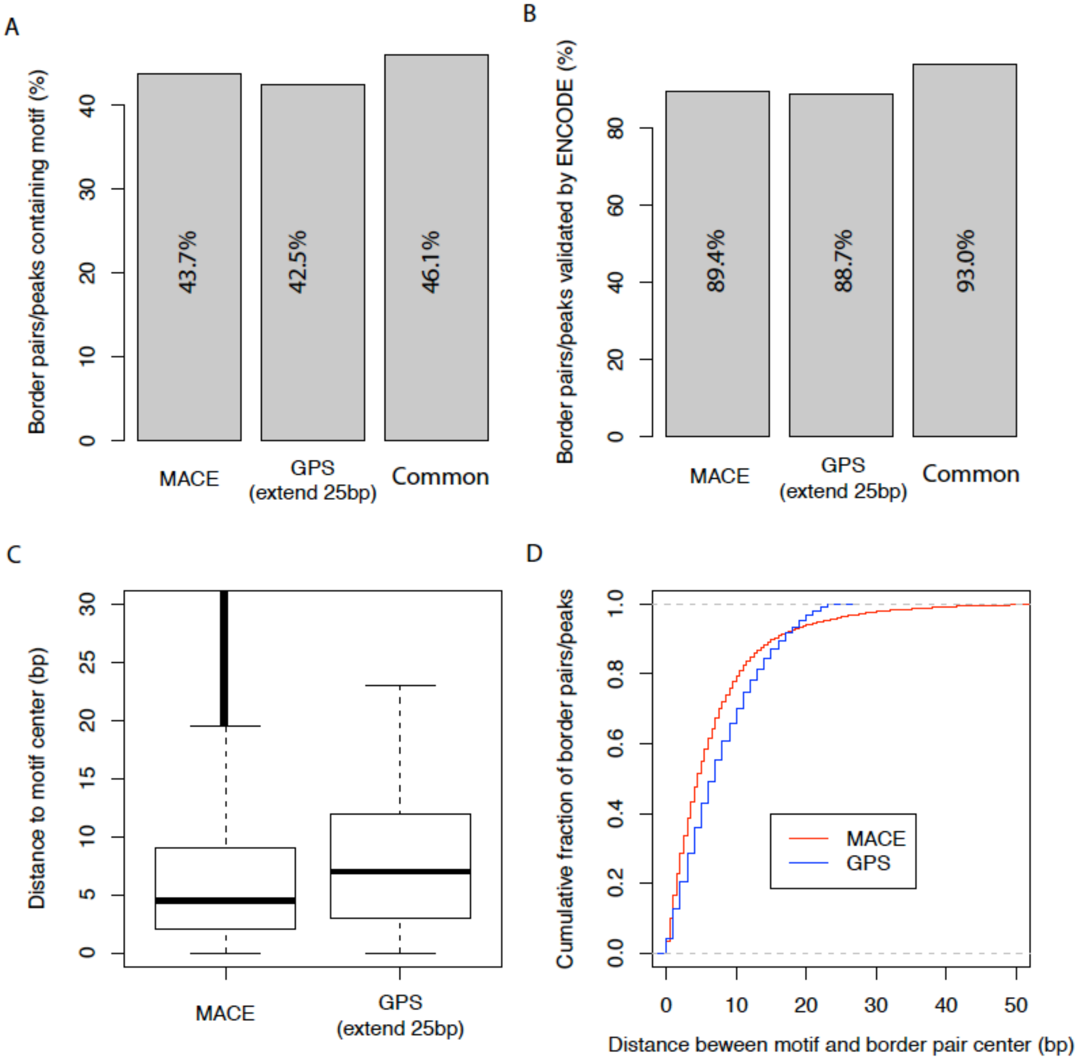

Supplementary Figure 13

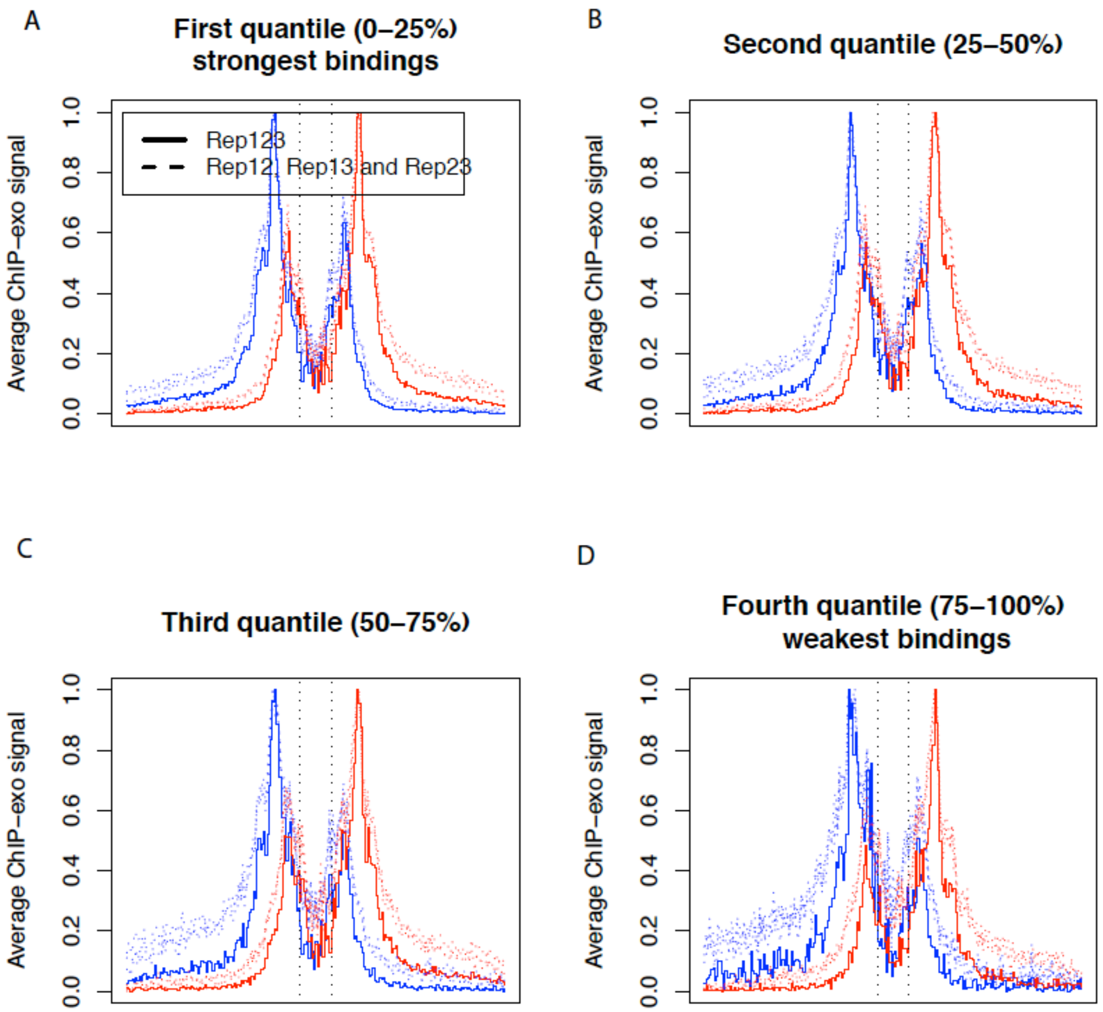

Supplementary Figure 14

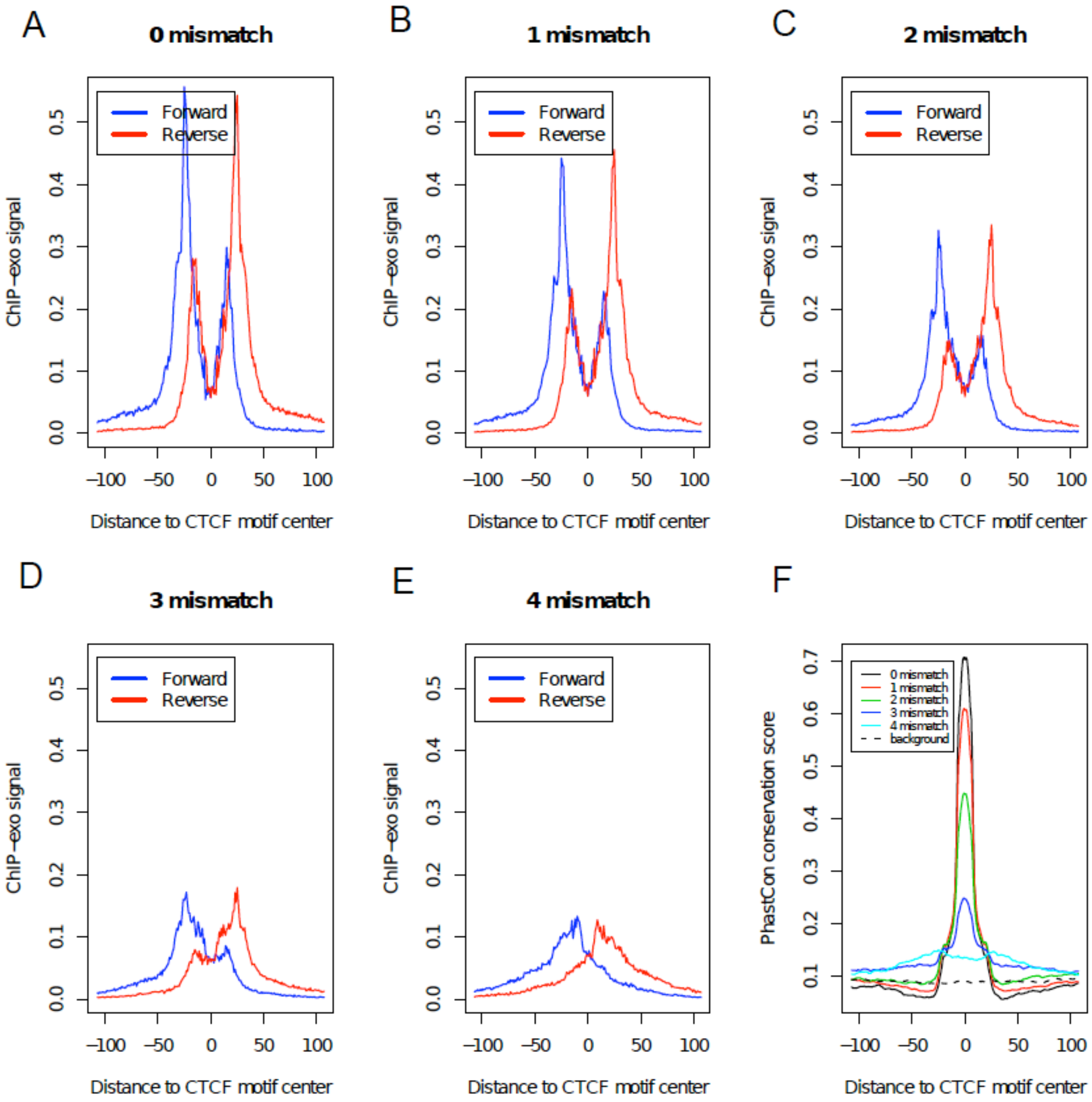

Supplementary Figure 15

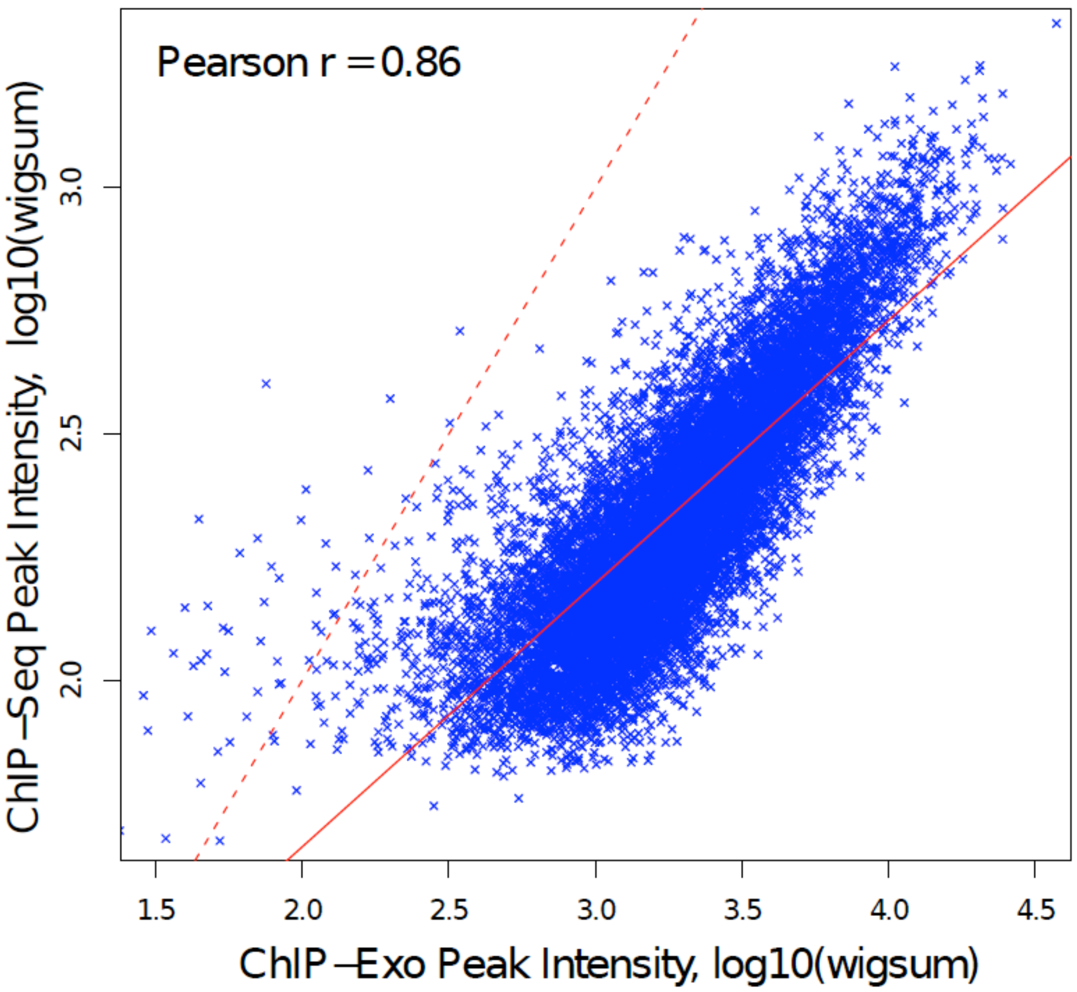

Supplement: SUPPLEMENTARY DATA [file supp_gku846_nar-00774-met-k-2014-File010.pdf]
